# Rare disruptive variants in the DISC1 Interactome and Regulome: association with cognitive ability and schizophrenia

**DOI:** 10.1038/mp.2017.115

**Published:** 2017-06-20

**Authors:** S Teng, P A Thomson, S McCarthy, M Kramer, S Muller, J Lihm, S Morris, D C Soares, W Hennah, S Harris, L M Camargo, V Malkov, A M McIntosh, J K Millar, D H Blackwood, K L Evans, I J Deary, D J Porteous, W R McCombie

**Affiliations:** 1 Stanley Institute for Cognitive Genomics, Cold Spring Harbor Laboratory, Cold Spring Harbor, NY, USA; 2 Department of Biology, Howard University, Washington DC, USA; 3 Centre for Genomic and Experimental Medicine, MRC/University of Edinburgh Institute of Genetics & Molecular Medicine, Western General Hospital, Edinburgh, UK; 4 Centre for Cognitive Ageing and Cognitive Epidemiology, Edinburgh, UK; 5 Institute for Molecular Medicine, Finland FIMM, University of Helsinki, Helsinki, Finland; 6 UCB New Medicines, One Broadway, Cambridge, MA, USA; 7 Genetics and Pharmacogenomics, MRL, Merck & Co, Boston, MA, USA; 8 Division of Psychiatry, University of Edinburgh, Royal Edinburgh Hospital, Edinburgh, UK; 9 Department of Psychology, University of Edinburgh, Edinburgh, UK

## Abstract

Schizophrenia (SCZ), bipolar disorder (BD) and recurrent major depressive disorder (rMDD) are common psychiatric illnesses. All have been associated with lower cognitive ability, and show evidence of genetic overlap and substantial evidence of pleiotropy with cognitive function and neuroticism. Disrupted in schizophrenia 1 (DISC1) protein directly interacts with a large set of proteins (DISC1 Interactome) that are involved in brain development and signaling. Modulation of DISC1 expression alters the expression of a circumscribed set of genes (DISC1 Regulome) that are also implicated in brain biology and disorder. Here we report targeted sequencing of 59 DISC1 Interactome genes and 154 Regulome genes in 654 psychiatric patients and 889 cognitively-phenotyped control subjects, on whom we previously reported evidence for trait association from complete sequencing of the *DISC1* locus. Burden analyses of rare and singleton variants predicted to be damaging were performed for psychiatric disorders, cognitive variables and personality traits. The DISC1 Interactome and Regulome showed differential association across the phenotypes tested. After family-wise error correction across all traits (FWER_across_), an increased burden of singleton disruptive variants in the Regulome was associated with SCZ (FWER_*across*_ *P*=0.0339). The burden of singleton disruptive variants in the DISC1 Interactome was associated with low cognitive ability at age 11 (FWER_*across*_ *P*=0.0043). These results identify altered regulation of schizophrenia candidate genes by DISC1 and its core Interactome as an alternate pathway for schizophrenia risk, consistent with the emerging effects of rare copy number variants associated with intellectual disability.

## Introduction

Schizophrenia (SCZ), bipolar disorder (BD) and recurrent major depressive disorder (rMDD) affect tens of millions of people worldwide. These disorders are moderately heritable and family history is a strong predictor of risk. Genome-wide association studies (GWAS), structural variant analyses and genome sequencing studies have identified that common single-nucleotide variants (SNVs), low penetrant rare SNVs, moderate to high penetrant copy number variants (CNVs) and potentially causal *de novo* mutations each play a role in the genetic etiology of SCZ and BD and, to a lesser extent, in rMDD.^1, 2, 3, 4^

There is now strong evidence for shared genetic risk across traditional diagnostic boundaries supporting the observation of ‘mixed’ diagnoses families.^5, 6^ GWAS studies capture common, ancient variation and point to an additive, polygenic architecture that transcends psychiatric diagnoses to predict cognitive ability variables.^1, 7, 8^ Lower cognitive function, both premorbid and post-onset, has been associated with these disorders, and recently polygenic risk score analysis has suggested a small, but significant, genetic correlation between risk for major mental illness and cognitive ability.^9^

In a complementary fashion to common variants identified in GWAS, rare variants have been identified that segregate with psychiatric disorder in a quasi-Mendelian manner and impact upon normal cognitive function.^10, 11^ One such example is a balanced t(1;11) (q42;q14) translocation in the *Disrupted in Schizophrenia 1* (*DISC1*) gene, which was identified in a large Scottish pedigree highly burdened with SCZ, BD and rMDD.^12^ Independent reports of linkage and association have since reported evidence for region-wide association of *DISC1* variants, or more commonly haplotypes, with these and other psychiatric disorders as well as for cognitive and neuropsychological traits.^13, 14, 15^ Although *DISC1* itself is not a GWAS significant finding, its interactor *PDE4B* and regulated gene *NRXN1* are reported as significant.^16, 17^ Convergent functional genomics approaches integrating the functional and genotypic data continue to support involvement of *DISC1* disruption in schizophrenia and related biological pathways.^18, 19^

Recently, we reported deep sequencing of the *DISC1* locus (528 kb) in 1542 samples that identified 2010 rare variants, of which ~60% were novel.^17^ We identified a common intronic variant with region-wide association for rMDD, and a rare missense mutation (R37W), previously reported in a SCZ case,^20^ in an individual with rMDD and in additional family members with mental disorders.^17^ Burden analysis also identified nominal associations with measures of depressed mood and cognitive ability at age 11, age 70 and cognitive ageing (change in cognitive ability between age 11 and 70).^17^ Motivated by these findings, we hypothesized that further insights might emerge from a directly comparable study of the DISC1 pathway genes.^21, 22, 23, 24^

Molecular studies have shown that DISC1 functions as a scaffold protein that is critical in cell signaling, neuronal development and ontogenesis through multiple protein-protein interactions.^25, 26^ DISC1-interacting partners (DISC1 Interactome) are enriched for proteins known to be involved in neural proliferation, migration, signaling and synaptic function.^14, 21, 22, 24, 27^ Positive case-control associations have been reported with psychiatric disorders for the following DISC1 Interactome genes: *ATF4*, *CIT*, *NDE1*, *PCM1*, *PDE4B*, *PDE4D* and *YWHAE*.^14^ In addition, structural rearrangements of *PDE4B* and *NDE1* have been reported in patients with SCZ.^28, 29^ Sequencing studies in SCZ have examined the burden of variants in *DISC1* and its interacting partners with mixed results. Moens *et al.*^30^ sequenced *DISC1* and ten interacting genes in 486 SCZ cases and 514 controls and observed an excess of rare missense variants in affected individuals. Kenny *et al.*^31^ sequenced the exons of 215 synaptic genes including *DISC1* and 22 interaction partners in 147 individuals with autism (ASD), 273 with SCZ and 287 controls. There was no enrichment of loss-of-function (LoF) mutations in the subset of DISC1-interacting genes, but singleton LoF variants were identified in *DISC1*, *DMD* and *TRAF3IP1* in patients with ASD. Although recent exome based studies of SCZ did not analyze a set of DISC1-interacting genes directly, an over representation of *de novo* and rare variation was observed in genes sets such as ARC-associated scaffold protein complex at the postsynaptic density.^32^ This finding suggests that the DISC1 Interactome may be enriched in genetic risk factors that function through perturbing neuronal development and function.^32^

In addition to the DISC1 Interactome, disruption of the expression of *DISC1*, or its interactors, has been shown to alter the expression of a further set of genes (DISC1 Regulome).^23, 33, 34^ This gene set is enriched for neurodevelopmental, synaptogenic and sensory perception genes as well as for registered drug targets for psychiatric and neurological disorders.^23^ We selected DISC1 Regulome genes on the basis of prior evidence of genetic association of common variants with psychiatric illness, as well as either: haplotype or model dependent expression patterns correlated with *DISC1* expression,^35^ *DISC1* alleles^23^ or with DISC1 Interactome genes; or proteins that directly interact with other Interactome gene proteins (Supplementary Information: gene selection).

Here we report the targeted sequencing of 59 DISC1 Interactome genes (including *TSNAX* and *DISC1*) and 154 DISC1 Regulome genes in the same cohort of subjects in which the full *DISC1* locus (528 kb) was sequenced and reported.^17^ As for our previous study, we report the gene-wide and gene-set burden analysis of rare variants and singletons in the DISC1 Interactome and Regulome with respect to psychiatric disorders, associated personality traits, and cognitive variables.

## Materials and methods

The materials and methods are described in full in the Supplementary Information. Briefly, we analyzed 1543 DNA samples comprising 654 cases (241 SCZ, 221 BD and 192 rMDD) from Scottish hospital patients and 889 community-dwelling, generally healthy older people from the Lothian Birth Cohort of 1936 (LBC1936), as described previously.^17^

A total of 213 genes were selected for sequence analysis (Table 1, Supplementary Information: gene selection, Supplementary Table S1). The *DISC1* locus (*DISC1*, *TSNAX* and *TSNAX-DISC1*) and 56 direct DISC1 protein-protein interactors defined the DISC1 Interactome gene set. A total of 154 additional genes related to *DISC1* expression from previous microarray analyzes comprised the DISC1 Regulome gene set. Genomic regions comprising ~11.7 Mb (3.3 Mb exons) were captured using a custom solution capture probe set (Roche NimbleGen, Pleasanton, CA, USA). Each sample capture was sequenced using a HiSeq2000 sequencer (Illumina, San Diego, CA, USA). Sequence reads were aligned to the human NCBI Build 36 (hg18) reference using BWA.^36^ Variant calling was performed using GATK,^37^ and high-quality SNVs were filtered by standardized filtering parameters. Using PLINK,^38^ we applied data quality control filters as described previously^17, 39^ to exclude samples and SNVs that introduce bias (Supplementary Figures S2–S5). Sanger sequencing was used to optimize the quality control filters and exclude all identified false positive SNVs from further analysis. SNVs were matched to hg19 coordinates using liftOver from UCSC, and ANNOVAR^40^ was used for variant annotation based on the human reference genome hg19 (RefSeq). The coding variants were grouped into three mutation classes, similar to previous analyses,^4, 32^ based on predicted functional effects: *Disruptive*, nonsense and splice site variants; NS_*strict*_, disruptive plus missense variants predicted as damaging by all five algorithms (SIFT,^41^ PolyPhen2 HumDiv and HumVar,^42^ LRT,^43^ and MutationTaster^44^); NS_*broad*_, disruptive plus missense variants predicted as damaging by at least one of the algorithms above; NS_*strict*_ is therefore a subset of NS_*broad*_. The burden and accumulation rate of rare variants (MAF<1%) and singletons in these mutation classes was assessed in each of the case cohorts and a combined cohort of all diagnoses; and for cognitive measures: cognitive ability at ages 11 and 70, change in cognitive ability, crystallized cognitive ability and general cognitive ability; and personality traits: neuroticism, anxiety and depression (Supplementary Information: phenotypes). The gene set and gene-wide burden analyses for all genes containing more than one rare variant were performed using the R package ‘SKAT’.^45^ Exact Poisson tests were performed in R to evaluate the accumulation rates of singletons and rare variants under a Poisson distribution in cases compared to controls in each functional mutation class. To control for multiple testing and reduce the risk of false positives, a bootstrap resampling approach (*n*=10 000) was used to estimate the significance of all tests (Family-Wise Error Rate, FWER), taking into account multiple tests within each diagnosis (FWER_within_) and across all diagnoses (FWER_across_)(Supplementary Information: rare variant burden analysis).

## Results

### Targeted sequencing and genetic discovery in 213 DISC1 Interactome and Regulome genes

A total of 1464 samples (95%) were sequenced to a minimum coverage depth of 20x across at least 80% of the targeted bases (Supplementary Table S2). Coverage was uniform across all sample groups (Supplementary Figure S1). Following sequence- and variant-based quality filters, 196 080 SNVs in 1446 samples (211 cases of SCZ, 169 cases of rMDD, 195 cases of BD, and 871 controls from the LBC1936) remained for further analyses (Supplementary Table S2 and Supplementary Figures S2–S5). Of the 196 080 SNVs, 78% have a MAF less than 1%. Only 40% are reported in the 1000 Genome Project European subset (Supplementary Table S3). On the basis of RefSeq functional annotations using ANNOVAR, 169 905 SNVs mapped to introns, 5410 to 3′ or 5′ UTRs, and 4523 to coding regions. Of the 4523 exonic variants, 1893 were functionally classified with respect to coding potential as silent variants, 2569 as missense, and 41 as nonsense. A further 24 SNVs were annotated as splice site variants. SNVs showing greater functional impacts on protein function are more likely to be rare: 100% of nonsense and 92% of splice site variants have MAF <1%, compared to 79% of silent and 78% of intronic variants.

### Analysis of genetic variation in the DISC1 Interactome and Regulome with psychiatric illness

#### Rare functional variant analysis in the DISC1 Interactome

There was no significant burden of rare disruptive, NS_*strict*_ or NS_*broad*_ variants in SCZ, BD, or rMDD nor in a combined cohort of all diagnoses compared to controls in the DISC1 Interactome (Supplementary Table S6). There was a nominal association of fewer rare disruptive variants in SCZ (unadjusted *P*=0.0188), but no significant difference between the accumulation rate of rare variants for any diagnosis after Family-Wise Error Rate (FWER) correction (Supplementary Table S7). None of the proportions of NS_*strict*_ and NS_*broad*_ rare or singleton variants deviated from the null hypothesis after FWER_across_ correction.

The gene-wide burden of non-synonymous coding changes was nominally, but not significantly increased in psychiatric disorders (unadjusted *P*=0.0048–0.0488) for several DISC1 Interactome genes. None survived correction for multiple testing (Supplementary Table S8).

#### Rare functional variant analysis in the DISC1 Regulome

**Figure 1:**
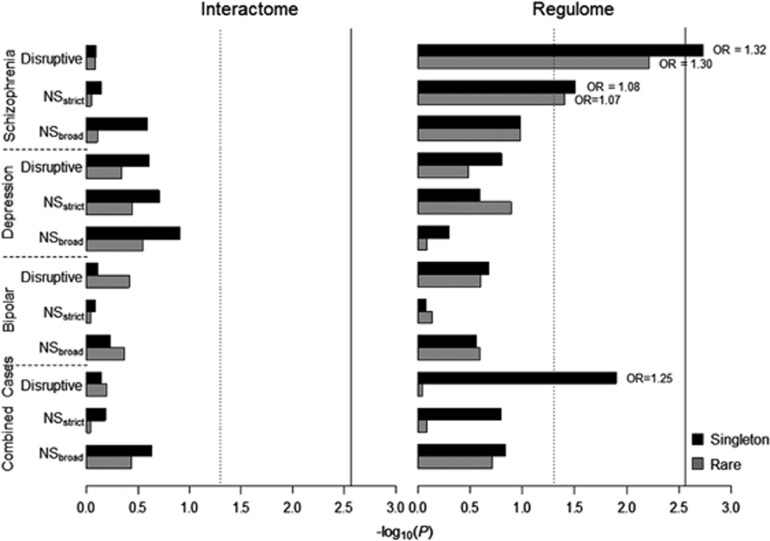
Gene set burden analysis of rare functional variants for case-control traits. Case-control gene set burden analysis of singletons and rare variants (MAF<1%) in the DISC1 Interactome (left) and Regulome (right). *x*-axis represents –log_10_(*P*), vertical dashed line: *P*=0.05, vertical solid line: FWER_*across*_ *P*=0.05; Odds ratio (OR) is labeled for the significant tests with *P*<0.05. Disruptive mutations, which included nonsense and splice site variants; NS_*strict*_, Non-synonymous strict damaging mutations which included disruptive variants plus missense variants predicted as damaging by all five algorithms (PolyPhen2 HumDiv and HumVar, SIFT, LRT and MutationTaster); NS_*broad*_, Non-synonymous broad damaging mutations which included disruptive plus missense variants predicted as damaging by at least one of the algorithms above.

We analyzed the burden and accumulation rates of rare and singleton functional variants in the DISC1 Regulome. For SCZ compared to control samples, we observed a significantly increased burden of singleton disruptive variants (unadjusted *P*=0.0019, FWER_within_ *P*=0.0069, FWER_across_ *P*=0.0339, OR=1.3162, SE=0.0941; Figure 1 and Supplementary Table S9), and a nominally higher accumulation rate (4.13-fold, unadjusted *P*=9.00 × 10^−4^, FWER_within_ *P*=0.0185, FWER_across_ *P*=0.0965, Supplementary Table S10). In addition, the accumulation rate of rare disruptive variants, as opposed to singleton disruptive variants, was 3.47-fold higher in SCZ cases than in healthy controls and remained significant after multiple test correction (unadjusted *P*=1.68 × 10^−6^, FWER_within_ *P*=1.00 × 10^−4^, FWER_across_ *P*=0.0022, Supplementary Table S10). Unlike singleton disruptive variants, although the burden of rare disruptive variants in SCZ was nominally significant, and survived FWER correction for all tests within the trait, it did not meet the threshold for tests across all traits (unadjusted *P*=0.0061, FWER_within_ *P*=0.0228, FWER_across_ *P*=0.0863, Supplementary Table S9). We also observed a nominally higher proportion and burden of NS_strict_ singleton and rare variants in SCZ and disruptive singleton and rare variants in combined cases compared to controls, but none survived FWER for all tests across all traits (Supplementary Tables S9 and S10). There was no evidence for an increased overall burden in rMDD, BD or combined cases compared to controls after FWER correction across all traits.

**Figure 2:**
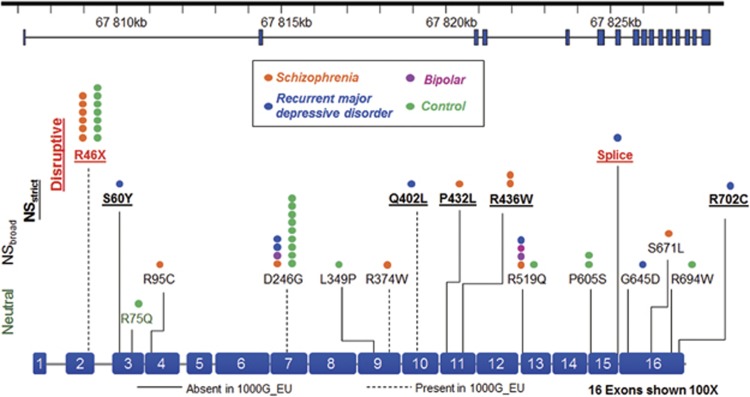
*Translin-associated factor X interacting protein 1* (*TSNAXIP1*) rare functional variants. *TSNAXIP1* exon structure and mutations. Definitions of Disruptive (red), NS_*strict*_ (underlined) and NS_*broad*_ mutations are given in Figure 1. Neutral variants (green) were defined as missense variants predicted as not damaging by any of the five predictive algorithms. Dash lines represent the variants present in the European subset of the 1000 Genomes Project (1000G_EU), and the solid lines represent the variants present in the 1000G_EU. The number of circles represents the number of samples carry the rare variant.

At the gene-wide level, *Translin-associated factor X interacting protein 1 (TSNAXIP1)* showed greater burden of NS_*strict*_ singletons in rMDD (unadjusted *P*=1.29 × 10^−4^, FWER_*within*_ *P*=0.0253) and NS_*strict*_ rare variants in SCZ (unadjusted *P*=2.22 × 10^−4^, FWER_*within*_ *P*=0.0410, Supplementary Table S11) compared to controls. However, these results did not survive correction for all tests (rMDD FWER_across_ *P*=0.0864, SCZ FWER_across_ *P*=0.1600). *TSNAXIP1* has 16 exons encoding 712 amino acids. We validated 17 rare coding variants in *TSNAXIP1* in all carriers, including 1 splice site, 1 nonsense and 15 missense variants (Figure 2 and Supplementary Table S12). Of these 17 rare substitutions, 4 were previously reported in the 1000 Genomes Project European subset. In total, 7 rare variants in *TSNAXIP1* including 2 disruptive and 5 predicted damaging missense variants contributed to the gene burden analysis of NS_*strict*_ variants in rMDD and SCZ. In a ‘leave-one-out’ approach, we determined that the nonsense mutation at chr16:66405794 (rs146214814, p.R46X) located in exon 2, contributed most to the higher burden of NS_*strict*_ variants in SCZ. Relative to controls, this disruptive variant had a 3.58-fold higher allele frequency in SCZ (0.0146 vs 0.0041) and was not observed in any other mental illness cohort. Further information on the neurobiology of TSNAXIP1 is given in Supplementary Information: TSNAXIP1.

### Burden analysis of coding variants on quantitative cognitive ability and personality traits associated with psychiatric illness

**Figure 3:**
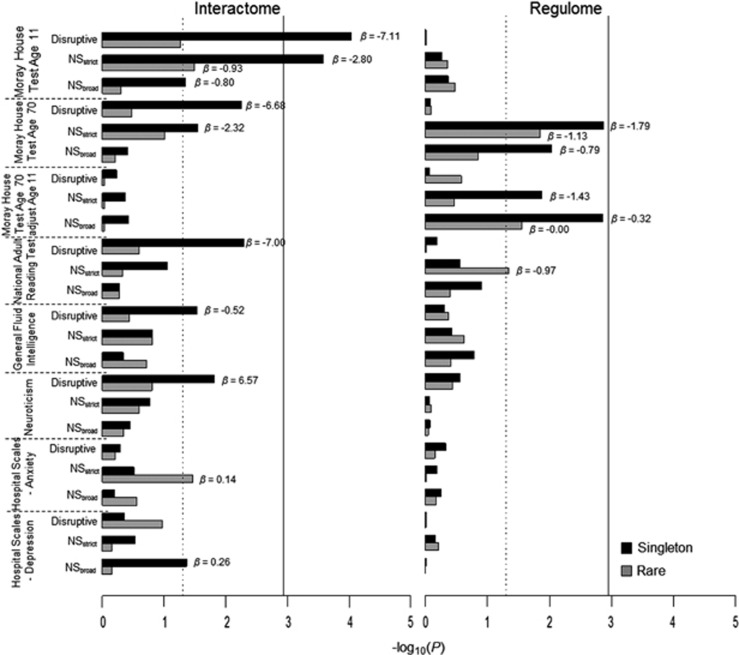
Gene set burden analysis of rare functional variants for quantitative traits. Quantitative trait gene set burden analysis of singletons and rare variants (MAF<1%) in the DISC1 Interactome (left) and Regulome (right). *x*-axis represents –log_10_(*P*), vertical dashed line: *P*=0.05, vertical solid line: FWER_*across*_ *P*=0.05; Effect size (*β*, Beta) is labeled for the significant tests with *P*<0.05. See phenotype descriptions in Supplementary Information for all quantitative traits. Moray House Test is the mental ability test used in the LBC1936 cohort. *A priori* hypothesis is that an increased burden of rare predicted damaging variants would reduce scores for cognitive variables and increase scores for personality traits. The definition of the variant classes is given in Figure 1.

We found that a significantly higher burden of singleton disruptive variants in the DISC1 Interactome was associated with lower cognitive ability assessed by Moray House Test (MHT) scores at age 11 (unadjusted *P*=9.35 × 10^−5^, FWER_*within*_ *P*=0.0005, FWER_*across*_ *P*=0.0043, *β*=−7.1141, SE=3.6863; Figure 3 and Supplementary Table S13). The burden of NS_*strict*_ singletons in the Interactome gene set was associated with lower MHT scores at age 11 (unadjusted *P*=0.0003, FWER_*within*_ *P*=0.0017, FWER_*across*_ *P*=0.0122, *β*=−2.7865, SE=1.2877). In addition, although these did not survive FWER_*across*_ correction, nominally significant associations in the burden of disruptive singletons were observed with MHT scores at age 70 (unadjusted *P*=0.0056, *β*=−6.6785), National Adult Reading Test (unadjusted *P*=0.0051, *β*=−6.9970) and General Fluid Intelligence (unadjusted *P*=0.0293, *β*=−0.5152). Interestingly, there were nominally significant associations between the burden of rare functional variants and increased symptoms of neuroticism (Disruptive singletons: unadjusted *P*=0.0154, *β*=6.5671), anxiety (NS_*strict*_ rare variants: unadjusted *P*=0.0349, *β*=0.1394) and depression (NS_*broad*_ singletons: unadjusted *P*=0.0431, *β*=0.2587). At the gene-wide level, no association was found between the variability in cognitive ability or personality scores and the burden of damaging or disruptive variants in any specific gene of the DISC1 Interactome after FWER_*across*_ correction (Supplementary Table S14).

In the analysis of the DISC1 Regulome, we observed a burden of NS_*strict*_ singletons associated with lower MHT scores at age 70 (unadjusted *P*=0.0014, *β*=−1.7895; Figure 3 and Supplementary Table S15) that withstood FWER correction for all tests within the trait (FWER_within_ *P*=0.0079), but not all tests across all traits (FWER_across_ *P*=0.0609). The burdens of NS_*strict*_ and NS_*broad*_ variants were nominally significantly associated with greater decrease in cognitive ability between the ages of 11 and 70 (NS_*strict*_ singletons: unadjusted *P*=0.0131, *β*=−1.4338; NS_*broad*_ singletons: unadjusted *P*=0.0014, *β*=−0.3175; NS_*broad*_ rare variants: unadjusted *P*=0.0280, *β*=−0.0010). At the gene-wide level, the strongest association with cognitive function was observed with rare and singleton NS_*strict*_ variants in *CACNA1C*, but this did not pass FWER_*across*_ correction (Supplementary Table S16).

## Discussion

Encouraging progress towards delineating the genetic architecture of psychiatric disorders has been made and roles for both common, rare and *de novo* mutations established. Rare variants of high impact can provide valuable mechanistic insight. Recent case-control deep sequencing studies indicate that in individuals with SCZ rare loss-of-function variants are enriched in genes related to synaptic function,^31^ in target genes of the FMPRP^32^ and in genes known to be associated with SCZ.^46^ The biological impacts of several *DISC1* missense variants identified through deep sequencing have been demonstrated.^30, 47^ We previously reported the discovery of rare disruptive *DISC1* variants in individuals with psychiatric illness and demonstrated the biological impact of the p.R37W variant.^17^ Here we report the association of both clinical diagnoses and cognitive ability with rare variants in the DISC1 Interactome and the DISC1 Regulome.

Before discussing these positive findings, we first consider some limitations of the study. Although the sample size was large by current standards, these numbers are modest in size for comprehensive rare variant detection.^17, 48^ We were unable to perform sex-specific analyses in our study given our sample size. Such analyses may be important in our understanding of the relationships between genetic variants and gene expression particularly in psychiatric illness, given reports of sex-specific differences in gene expression in the brain^49, 50^ but also due to reports of sex-specific differences in association of variants and haplotypes in *DISC1*,^51, 52, 53^ the success of the CONVERGE strategy that relied on mapping loci for severe depression within a female-only cohort^54^ and the differences in disease presentation between sexes that have likewise been reported.^55^ Burden analysis increases the power of analyses in such small samples, but the rules for annotating rare variants as ‘damaging’ are far from foolproof: biological validation is required. Last, but not least, whole genome sequencing of all 1543 individuals, while ideal, was beyond the scope of our resources. Targeted capture sequencing was a practical option, but it is likely that relevant variants will have been missed by virtue of poor capture. It is also almost certainly the case that our list of *bona fide* DISC1 interactors is incomplete, and that contra wise, not all members of the Regulome that met our inclusion criteria will be regulated by DISC1 in practice.

Acknowledging these limitations, there were findings of note. No association was seen between rare variants in the DISC1 Interactome and any psychiatric diagnosis. There was, however, a significant excess of singleton disruptive variants in the DISC1 Regulome associated with SCZ, but not with BD or rMDD. We have shown that disruptive and NS_*strict*_ singleton variants in the DISC1 Interactome show significant association with cognitive ability at age 11. These classes of variants are also nominally associated in the DISC1 Regulome with cognitive ability at age 70 and change in cognitive ability between age 11 and 70. The DISC1 Regulome gene set was assembled from genes that show both i) altered expression in response to genetic variation in *DISC1* or its interactors, or are themselves protein interactors of the core complex, and ii) evidence of association with psychiatric illness from candidate gene studies, or some of the earliest genome-wide association studies.^1, 56, 57^ We note that in this study, we found nominal association of rare Regulome variants with both increased schizophrenia risk and lower adult cognitive ability, particularly in older age. This mirrors the observation of association with common variants from the DISC1 Regulome in GWAS studies.^8, 9, 58, 59^ Overall, the patterns of associations seen across diagnostic and cognitive traits in the DISC1 Interactome and Regulome are consistent with the hypothesis that genetic disruption of DISC1 or its direct interactors has a proximal effect on cognitive ability and a distal effect, through regulation of gene expression, on schizophrenia risk in later life. Indeed, we have shown previously that disruption of the *Disc1* gene in mice results in altered expression of *Nrxn1*,^33^ a gene in which copy number variation, common variants and rare variants are associated with schizophrenia.^60, 61, 62^ The hypothesis of a distal effect of variants in the Interactome on disease risk is also consistent with the recent association of copy number variants linked to intellectual disability with schizophrenia in a much larger sample.^63^

Four genes in the Interactome gene set, *DISC1*, *CIT*, *DST* and *MAP1A*, were nominally associated at the gene level with cognitive ability at age 11 (Table 1). CIT and MAP1A are known interactors with DLG4, PSD-95, which in turn interacts with the cytoplasmic tail of NMDA receptor subunits and with shaker-type potassium channels, regulating the ratio of excitatory to inhibitory synapses in the hippocampus.^64, 65, 66^ Because of the complex network multiplicity of protein interactions identified by the DISC1 Interactome gene set, we cannot conclude that our association with cognitive ability at age 11 is specific, or restricted to, the DISC1 Interactome or indeed that this phenotype is the only one likely to be associated with rare functional variants in this set of genes. This important caveat also applies not only to the DISC1 Regulome gene set, in which 16 genes were nominally associated at the gene level with schizophrenia (Table 1), but likely to the majority of brain-expressed gene sets. None of the gene level associations survived FWER correction across all tests.

To better understand the distinctive patterns of association between the DISC1 Interactome and Regulome, and their relationship to previously published mental health-related gene sets, we performed gene ontology (GO) enrichment analyses (Supplementary Tables S17, 18 and Supplementary Figures S9). The DISC1 Interactome is significantly enriched for proteins involved in regulation of nervous system development, microtubule cytoskeleton organization, and vesicle localization (Supplementary Tables S17, 18 and Supplementary Figures S9). Our findings suggest that disruptive singletons in these biological processes may make significant contributions to variability in cognitive function. These processes have also been associated with intellectual disability.^67^ Together, these datasets suggest that there is a spectrum of effect sizes or penetrance associated with genetic variants in this pathway. In contrast, the DISC1 Regulome is enriched for genes involved in synaptic transmission and glutamate-gated ion channel activity, reflecting the regulation by the DISC1 Interactome of these processes and their importance as inferred from GWAS. The specificity of the association between single disruptive Regulome variants and SCZ in our sample suggests a greater impact of glutamate dysregulation in this disorder than BD or rMDD. A role for DISC1 in glutamate-related processes has previously been suggested in both a mouse model and in the t(1;11) translocation family.^68, 69^ Comparison of the GO terms associated with both the DISC1 Interactome and Regulome reveals largely independent GO term associations with a very limited set of intersecting terms focused on negative regulation of cellular process, protein binding, and cell projections (Supplementary Figures S9).

In conclusion, and despite the limitations, these findings provide further genetic evidence to support the impact of both DISC1-interacting proteins and genes whose expression is modulated by genetic variants in the DISC1 pathway on schizophrenia.

## Figures and Tables

**Table 1:** DISC1 (Number 1) Interactome and Regulome

*DISC1 Interactome*		*DISC1 Regulome*				
*AKAP9*	*KCNQ1*	*ACTG2*	*CYB5R2*	*HNRNPK*	*NSUN6*	*SORT1*
*AP4B1*	*KIF3A*	*ADCY1*	*DLG2*	*HOOK3*	*NUDT1*	*SOX10*
*AP4M1*	*KIFAP3*	*ADCY3*	*DLGAP1*	*HSPA2*	*NUMB*^a^	*SRSF6*
*APP*	*MACF1*	*AKT1*	*DMRT2*	*ID1*	*OGFR*	*STAT5A*
*ATF4*	*MAP1A*^b^	*ANKRD16*	*DTNBP1*	*IL9R*	*OLIG2*	*SV2B*
*ATF5*	*NDE1*	*APPL2*	*DUSP6*	*ITPR1*	*OPTN*	*SYN1*
*ATF7IP*	*NDEL1*	*ARC*	*DYNLL1*	*JUNB*	*PAK3*	*SYN2*
*CCDC88A*	*OLFM1*	*ARHGEF11*	*EEF1E1*	*KIF5C*^a^	*PCBP1*	*SYP*
*CDC5L*	*PAFAH1B1*	*ATP6V0A1*	*EEF2K*	*LIMA1*	*PCM1*^a^	*TACR1*
*CDK5RAP3*	*PCNT*	*BANK1*	*EGR2*	*LITAF*	*PDE4A*	*TGFBI*
*CEP290*	*PDE4B*	*BBS4*	*EGR3*	*LPIN1*	*PITPNM1*	*THOP1*
*CIT*^b^	*PDE4D*	*BCR*^a^	*EGR4*	*MARCH3*	*POU6F1*	*TIMM8A*
*CLIC1*	*RAD21*	*BDNF*	*ERBB2*	*MCPH1*	*PPARGC1A*	*TMEM163*
*CLU*	*RANBP9*	*BEX2*	*ERBB3*	*MDFIC*	*PPP4R2*	*TNFAIP8L2*
*CTNNB1*	*SH3BP5*	*BLZF1*	*ERBB4*^a^	*MEG3*	*PRKCB*	*TNFRSF11B*
*DCTN1*	*SMC3*	*BRD1*^a^	*FHIT*	*MGLL*	*PRR18*	*TP53RK*
*DCTN2*	*SNX6*	*C5orf48*	*GABBR1*	*MMP7*	*PRRC2A*	*TRIOBP*
*DISC1*^b^	*SYNE1*	*CACNA1C*	*GABRA5*	*MTPAP*	*RAB3A*	*TSNAXIP1*^a^
*DIXDC1*	*TNIK*	*CALM3*	*GJA5*^a^	*MYRIP*	*RGS4*	*TUBB3*
*DPYSL3*	*TRAF3IP1*	*CAMK1D*	*GNB1L*	*NAPB*	*RHOA*	*UBL3*
*DST*^b^	*TRIO*	*CAMK4*	*GRIA1*	*NCOR2*	*RPS6KA2*	*UQCRC1*^a^
*EIF3H*	*TSNAX*	*CCND2*	*GRIA2*^a^	*NDN*	*SCHIP1*	*USP7*
*EXOC4*	*TSNAX-DISC1*	*CCNE1*	*GRIA3*	*NEK1*	*SERPINI1*	*VASH2*
*FBXO41*	*TUBB*	*CETP*	*GRIN1*	*NFKB2*^a^	*SKAP1*	*VPS45*
*FEZ1*	*TUBG1*	*CHMP1A*	*GRIN2A*^a^	*NFKBIA*	*SLC17A7*	*VRK2*
*GNB1*	*WNT3A*	*CHRNA5*	*GRIN2B*	*NOS1*	*SLC1A2*^a^	*WDFY1*
*GRB2*	*YWHAE*	*CLINT1*	*GRM3*	*NOS1AP*	*SLC41A2*	*XPOT*
*GRIPAP1*	*YWHAZ*	*COMMD1*	*HERPUD1*	*NOTCH4*	*SMC1A*	*ZHX2*
*GSK3B*		*CORO1B*	*HIPK2*	*NRG1*	*SNAP91*	*ZIC1*^a^
*ITSN1*		*CPLX2*	*HIST1H2BN*	*NRXN1*^a^	*SNPH*	*ZNF140*
*KALRN*		*CTTN*	*HLA-DQA1*^a^	*NRXN3*	*SORCS3*	

a Genes with a nominally significant burden p-values for schizophrenia (16 of 154 genes in the DISC1 Regulome). These gene level results did not survive family-wise error rate correction across all tests.

b Genes with a nominally significant burden p-values for cognitive ability at age 11 (4 of 59 genes in the DISC1 Interactome).
