# Supplementary material for: Rare disruptive variants in the DISC1 Interactome and Regulome: association with cognitive ability and schizophrenia
Source: Mol Psychiatry. 2017 Jun 20;23(5):1270–7. doi: 10.1038/mp.2017.115 (PMC5984079; doi:10.1038/mp.2017.115)
Supplement: Supplementary Information [file mp2017115x1.doc]

**Supplementary Information: Rare disruptive variants in the DISC1 Interactome and Regulome: association with cognitive ability and schizophrenia. Teng *et al*.**

[Sample Collection 3](#__RefHeading___Toc474478494)

[Phenotypes 3](#__RefHeading___Toc474478495)

[Gene Selection 5](#__RefHeading___Toc474478496)

[Targeted Resequencing 7](#__RefHeading___Toc474478497)

[Variant Calling 8](#__RefHeading___Toc474478498)

[Case-Control Quality Control 9](#__RefHeading___Toc474478499)

[Variant Annotation 11](#__RefHeading___Toc474478500)

[Variant Validation and Evaluation 12](#__RefHeading___Toc474478501)

[Rare Variant Burden Analysis 13](#__RefHeading___Toc474478502)

[GO Enrichment Analysis 15](#__RefHeading___Toc474478503)

[TSNAXIP1 16](#__RefHeading___Toc474478504)

[URLS 18](#__RefHeading___Toc474478505)

[Supplementary References 19](#__RefHeading___Toc474478506)

[Supplementary Tables 26](#__RefHeading___Toc474478507)

[**Supplementary Table S1: DISC1 Interactome and Regulome Gene List** 26](#__RefHeading___Toc474478508)

[**Supplementary Table S2: Sequencing Summary Statistics** 27](#__RefHeading___Toc474478509)

[**Supplementary Table S3: Variant Summary Statistics** 28](#__RefHeading___Toc474478510)

[**Supplementary Table S4: Sensitivity and Specificity of DISC1 Variant Discovery by Capture Sequencing** 29](#__RefHeading___Toc474478511)

[**Supplementary Table S5: Summary of Validated Disruptive Variants** 30](#__RefHeading___Toc474478512)

[**Supplementary Table S7. Gene Set Exact Poisson Tests of Rare Functional Variants in the DISC1 Interactome for Case-Control Traits.** 33](#__RefHeading___Toc474478513)

[**Supplementary Table S8. Gene-Wide Burden Analysis of Rare Functional Variants in the DISC1 Interactome for Case-Control Traits** 34](#__RefHeading___Toc474478514)

[**Supplementary Table S9. Gene Set Burden Analysis of Rare Functional Variants in the DISC1 Regulome for Case-Control Traits** 35](#__RefHeading___Toc474478515)

[**Supplementary Table S10. Gene Set Exact Poisson Tests of Rare Functional Variants in the DISC1 Regulome for Case-Control Traits** 36](#__RefHeading___Toc474478516)

[**Supplementary Table S11. Gene-Wide Burden Analysis of Rare Functional Variants in DISC1 Regulome for Case-Control Traits** 37](#__RefHeading___Toc474478517)

[**Supplementary Table S12. Translin-Associated Factor X Interacting Protein 1 (*TSNAXIP1*) Rare Mutations** 38](#__RefHeading___Toc474478518)

[**Supplementary Table S13. Gene Set Burden Analysis of Rare Functional Variants in the DISC1 Interactome for Quantitative Traits** 39](#__RefHeading___Toc474478519)

[**Supplementary Table S14. Gene-Wide Burden Analysis of Rare Functional Variants in the DISC1 Interactome for Quantitative Traits** 41](#__RefHeading___Toc474478520)

[**Supplementary Table S15. Gene Set Burden Analysis of Rare Functional Variants in the DISC1 Regulome for Quantitative Traits** 42](#__RefHeading___Toc474478521)

[**Supplementary Table S16. Gene-Wide Burden Analysis of Rare Functional Variants in the DISC1 Regulome for Quantitative Traits** 44](#__RefHeading___Toc474478522)

[**Supplementary Table S17. Gene Ontology Enrichment Analyses of the DISC1 Interactome** 45](#__RefHeading___Toc474478523)

[**Supplementary Table S18. Gene Ontology Enrichment Analyses of the DISC1 Regulome** 46](#__RefHeading___Toc474478524)

[Supplementary Figures 47](#__RefHeading___Toc474478525)

[**Supplementary Figure S1: Distribution of Coverage in Samples** 47](#__RefHeading___Toc474478526)

[**Supplementary Figure S2: A Flowchart of Quality Control** 48](#__RefHeading___Toc474478527)

[**Supplementary Figure S3: Multidimensional Scaling Plots** 49](#__RefHeading___Toc474478528)

[**Supplementary Figure S4: Missing Variant Rate versus Heterozygosity Rate across all Samples** 50](#__RefHeading___Toc474478529)

[**Supplementary Figure S5: Missing Sample Rate versus Hardy-Weinberg equilibrium *P*-values across all SNVs** 51](#__RefHeading___Toc474478530)

[**Supplementary Figure S6: Minor Allele Frequencies in the 1000 Genomes Project and LBC1936 controls** 52](#__RefHeading___Toc474478531)

[**Supplementary Figure S7: Quantile-Quantile Plots of Gene-wide Burden Analysis of Rare Damaging Mutations for Case-Control Traits** 53](#__RefHeading___Toc474478532)

[**Supplementary Figure S8: Quantile-Quantile Plots of Gene-wide Burden Analysis of Rare Damaging Mutations for Quantitative Traits** 54](#__RefHeading___Toc474478533)

[**Supplementary Figure S9: Gene Ontology Enrichment Analyses for Biological Process** 55](#__RefHeading___Toc474478534)

[**Supplementary Figure S10: Gene Ontology Enrichment Analyses for Molecular Function** 56](#__RefHeading___Toc474478535)

[**Supplementary Figure S11: Gene Ontology Enrichment Analyses for Cellular Component** 57](#__RefHeading___Toc474478536)

**Sample Collection**

The ascertainment of samples for this study, that had been approved by the Research Ethics Committee, has been described previously.^1^ Samples for this study were collected from the Scottish participants. All patients provided informed consent for the use of their data and samples for medical research. Case samples were unrelated schizophrenia (SCZ), bipolar disorder (BD) and recurrent major depressive disorder (rMDD) hospital patients diagnosed based on DSM-IV criteria as described previously.^2^ The Lothian Birth Cohort of 1936 (LBC1936) samples, which had quantitative measures of mood and cognitive aging, were used as the controls in this study.^3,4^ The LBC1936 includes 1 091 community-dwelling individuals without dementia (548 males and 543 females), residing in or around the city of Edinburgh, Scotland.^3,5^ The majority of the LBC1936 had participated in the Scottish Mental Survey 1947 at a mean age of 10.9 years and then at a mean age of 69.5 years (standard deviation=0.8) in a follow-up assessment approximately 59 years later. These assessments are referred to as ages 11 and 70 throughout.

In total, 1 543 samples, including 241 cases of SCZ, 221 cases of BD, 192 cases of rMDD and 889 controls from the LBC1936 were sequenced in the present study.

**Phenotypes**

**Clinical diagnoses**

Affected individuals were inpatients or outpatients of hospitals in South East or South Central Scotland. Subjects were interviewed by an experienced psychiatrist and a venous blood sample was given for DNA extraction. Diagnoses were made according to Diagnostic and Statistical Manual (DSM)-IV criteria^6^ based on case note review and personal interview using The Schedule for Affective Disorders and Schizophrenia – lifetime version (SADS-L).^7^ Final diagnoses were reached by consensus between two experienced psychiatrists.

**Cognitive variables - LBC1936**

The majority of LBC1936 participants undertook the Moray House Test (MHT) at about age 11 years.^3^ They retook the same MHT at about age 70. The MHT is a group-administered, paper-and-pencil test that has a time limit of 45 minutes. The questions are mainly verbal reasoning items with some arithmetical and abstract items. MHT scores were converted into an IQ-type scale, with a mean of 100 and standard deviation of 15.^8^ Cognitive change between ages 11 and 70 were calculated by adjusting each test for age at testing and then regressing age 70 MHT on age 11 MHT and using the unstandardized residual. A General Fluid (gf) Intelligence at age 70 was derived from principal components analysis of 6 Wechsler Adult Intelligence Scale–III UK nonverbal subtests^9^ (Matrix Reasoning, Letter Number Sequencing, Block Design, Symbol Search, Digit Symbol, Digit Span Backward), as described previously.^10^ Crystallized intelligence at age 70 was measured using the National Adult Reading Test (NART).^11^ Cognitive measures were adjusted for age at testing and sex.

**Mood and personality trait - LBC1936**

The mood states of anxiety and depression were assessed using the Hospital Anxiety Depression Scale (HADS).^12^ The personality trait of neuroticism were measured using the NEO Five-Factor Inventory.^13^ Personality measures were adjusted for age at testing and sex.

**Gene Selection**

A total of 213 genes that directly or indirectly interact with DISC1 were selected for targeted resequencing in the study Genes were grouped into following categories.

1. **DISC1 Interactome**: A set of 59 genes consisting of *DISC1* locus (*Disrupted in Schizophrenia 1* (*DISC1*); *Translin-associated factor X* (*TSNAX*); *TSNAX-DISC1 readthrough*) and direct DISC1 Protein-Protein Interacting (PPI) genes based on annotations in the protein interaction databases IntAct and Mentha.^14,15^
2. **DISC1 Regulome**: 154 genes based on the following criteria:

a) prior evidence of genetic association with psychiatric illness (GWAS, CNV and candidate genes studies.^16–18^

b) expression altered in hippocampus of DISC1 mutant mice.^19^

c) expression patterns dysregulated in lymphoblastoid cell lines of DISC1 t(1:11) translocation carriers (see below).

d) expression regulated by risk variants in DISC1 or DISC1 interactors.^20^

e) proteins that directly interact with other Interactome gene proteins.

All genes selected were required to meet criterion a) and one other from b-e. The full list of 213 DISC1 Interactome plus Regulome genes targeted for this study are listed in in Supplementary Table 1.

**Differential gene expression in lymphoblastoid cell lines of t(1;11) translocation carriers**

This experiment has been published as part of a PhD Thesis. Full details can be found in Briggs, Gareth James, “Investigating putative pathogenic mechanisms within a family in which a chromosomal translocation confers risk of major mental illness”. PhD Thesis, University of Edinburgh ([www.era.lib.ed.ac.uk](http://www.era.lib.ed.ac.uk/)). Gene expression from Epstein-Barr virus transformed lymphblastoid cell lines was assessed using a proprietary Rosetta chip in a study implemented by Maerk Sharp Dohme. Gene expression was detected for 10 923 genes. Differential expression was analysed between translocation carriers and family controls with techinical replicates (fold change ±1.3 and *P*≤0.05). 1 010 genes were differentially expressed (9.2%) and functional enrichment analyses showed a clear signature for cell cycle and DNA replication as well as possible effects on immune function and inflammation pathways.

**Target Design**

A custom solution capture probe set (Roche NimbleGen) was developed to target approximately 11.7Mbp (0.38%) of the human genome (hg18) representing the exons (3.3 Mbp) and promoters of all 213 DISC1 Interactome plus Regulome genes as well as conserved regions 20kb upstream, downstream and across each gene. The exon coordinates of every isoform of each gene were extracted from the RefSeq and UCSC Gene lists (hg18). The promoters were defined as 2kb upstream regions of transcription start site of all selected genes. The conserved regions were defined as runs of at least 10bps with an average score ≥0.3 using phastCons 44-vertebrate alignment.^21^ Expanding our analysis to include 100bp flanking each of the 67 551 targets represents 21.5 Mbp (0.70%) of the human genome, taking into account overlaps between flanking targets.

**Targeted Resequencing**

Genomic DNA (1ug) extracted from blood samples of each individual was sheared to create fragments with an average size of 250bp using a Covaris S2 sonicator system (temperature: 4°C, duty cycle: 10%, intensity: 5, cycles per burst: 200, time: 90 s). The fragment size distribution of the DNA was checked using a DNA 1000 Bioanalyzer chip (Agilent Technologies), and the DNA was concentrated using Agencourt Ampure XP beads (Beckman Coulter). The NEXTflex DNA Sequencing Kit (Bioo Scientific) was used for sequencing library preparation including end repair, size selection (~400bp insert size), adenylation, and adaptor ligation. The DNA was purified using Ampure XP beads, and amplified using LM-PCR. The DNA was then quantified using a Quant-iT fluorometric assay and the Agilent Technologies 2100 Bioanalyzer DNA 1000 Kit. The library was hybridized with the custom solution capture probes described above. The hybridization was performed, according to the NimbleGen SeqCap User’s Guide, for 72 hours at 47°C with the barcode-blocking oligo. The captured DNA was recovered using Streptavidin Dynabeads. The post-capture sample library amplifications were performed using two LM-PCR reactions per sample to reduce PCR bias followed by an Ampure XP bead clean-up. The post-capture PCR enriched library was quantified by using a qPCR library quantification kit (KAPA Biosystems). The library fragment distribution was determined using a High Sensitivity DNA kit on the Agilent Bioanalyzer system, and the results suggested that the samples have broad peaks ranging from 250bp to 850bp with the highest peak at around 400bp. The sequencing flow cells were generated using a CBot reagent plate (Illumina), in total 16 barcoded samples were sequenced across three lanes of a flowcell using HiSeq2000 (Illumina) with paired-end 101 reads. In total 1464 (95%) samples with 80% of the targets at ≥20x read depth were used in the downstream analysis, and the other 79 samples were excluded from the further analyses. The average mean target coverage of the samples was 116x read depth (Supplementary Table S2), and no evidence of significant sequencing bias was observed between the case and control groups (Supplementary Table S2 & Supplementary Figure S1).

**Variant Calling**

Variant calling was performed using our standardized variant calling pipeline. Paired-end reads were aligned to the human NCBI Build 36 (hg18) reference using BWA.^22^ SAMtools^23^ was used to convert to bam format, sort, index and merge the aligned sequencing files. Picard was applied to remove duplicate read pairs, which were an artifact of the PCR amplification during sample preparation. BamTools^24^ was used to filter for properly paired reads and for mapping quality of >=20. The Genome Analysis Toolkit (GATK)^25^ was used to recalibrate base quality scores, realign indel regions and call single nucleotide variants (SNVs) and small insertions and deletions (INDELs) located in the targets as well as in the 100bp flanking regions of each target. The SNVs located in called INDEL regions were masked. Standardized filtering parameters (minimum mapping quality 40, minimum confidence score 30, minimum depth 6, clusterWindowSize 10, clusterSize 3) were applied to select the SNVs with high confidence. Additional filters used in SNV calling included "HaplotypeScoreFilter" (HaplotypeScore>13.0), "QDFilter" (QD<2.0), "FSFilter" FS>60.0 MQRankSumFilter (MQRankSum<-12.5) and "ReadPosRankSumFilter" (ReadPosRankSum<-8.0). To establish the filtering criteria for SNVs with high quality, we compared the SNV calling results with our previous Sanger sequencing validations^1^ in the TRAX/DISC1 region. The SNVs with filters “PASS” or “HaplotypeScoreFilter” were selected for further analysis, and showed good agreement with 100% and 98% sensitivity for detecting the true exonic and rare SNVs in the TRAX/DISC1 region. VCFtools^26^ was used to merge the VCF files and convert from VCF to PLINK format (ped and map files), and multi-allelic SNVs were excluded for further analysis. GATK^25^ was also used to generate “coverage” VCF files including calls at all sites from the recalibrated bam files. An in-house Perl script was applied to extract the genotypes of reference allele homozygotes from the VCF files to fill missing information in the merged VCF file, and the genotypes were set as missing if they had a coverage of <6X.

**Case-Control Quality Control**

To reduce the number of potential false-positive and false-negative associations, we performed several data quality control analyses using PLINK^27,28^ to remove poor quality samples and SNVs. The detailed quality control strategy is illustrated in Supplementary Figure S2.

**Sample Quality Control**

To minimize issues with population stratification that can confound case-control studies, we performed multidimensional scaling (MDS) analysis of our samples together with genotype data from HapMap (release 23). We removed four samples that deviated from the Hapmap European population and the samples sequenced in this study (Supplementary Figure S3).

1. To exclude samples of low quality,^28^ we removed three samples with a missing genotype rate>7.5% (Supplementary Figure S4, vertical dashed line) and three samples with an outlying heterozygosity rate ± 4 s.d. from the mean (Supplementary Figure S4, horizontal dashed lines),
2. Two samples with inconsistency between chrX heterozygous SNV rates and reported gender were removed.
3. Finally, three samples with cryptic relatedness (PI_HAT>0.3) identified based on pairwise Identity by Descent (IBD) using PLINK default settings were excluded.

**SNV Quality Control**

Following the removal of poor quality and unreliable samples, SNVs were filtered as follows to minimize issues with coverage, and genotyping errors:

SNVs with a genotype missing rate >0.2 were removed (Supplementary Figure S5, vertical dashed line).

SNVs with a Hardy-Weinberg equilibrium P-value <0.00001 (Supplementary Figure S6, horizontal dashed lines) were removed.

SNVs with a minor allele frequency equal to zero due to sample elimination were excluded.

Finally singleton variants with high strand bias ("FSFilter" FS>30.0) were excluded as these singletons showed high false positive rate in validation.

**Variant Annotation**

The SNVs were matched to hg19 coordinates using liftOver from UCSC. ANNOVAR^29^ was used to predict the mutational class (exon, splice site, nonsense, missense, silent, UTR, etc.) of each variant based on RefSeq (hg19). ANNOVAR uses the precedence (exonic=splicing>ncRNA>UTR5/UTR3>intron>upstream/downstream>intergenic) to decide what function to print out when a variant fit multiple functional categories. Analysis was restricted to variants located within 5kb upstream and downstream of each gene. ANNOVAR was used to identify the SNVs that present in dbSNP144 and determine the allele frequency of SNVs in the 1000 Genomes Project (2015aug version). Rare variants were classified as SNVs with a minor allele frequency (MAF) <1% in the combined case-control samples, and singleton variants were SNVs observed in only one sample. The functional effect of each variant was predicted based on five *in silico* algorithms (SIFT^30^, PolyPhen2 HumDiv and HumVar^31^, LRT^32^ and MutationTaster^33^) using ANNOVAR. We applied a strategy similar to that used in previous studies^34,35^ to classify the coding variants into three damaging mutation classes: (1) disruptive mutations including nonsense and splice-site variants; (2) non-synonymous strict damaging mutations (NS*_strict_*) including disruptive variants plus missense variants predicted as damaging by all five algorithms above; and (3) non-synonymous broad damaging mutations (NS*_broad_*) including disruptive plus missense variants predicted as damaging by at least one of the five algorithms listed above.

**Variant Validation and Evaluation**

We used Sanger sequencing to validate variants. PCR primers flanking the SNV regions were picked by Primer3 and manufactured by Sigma. LongAmp PCR was used to amplify the sample DNA. Sanger sequencing was performed using the primers and the Big Dye terminator sequencing kit (Life Technologies) on an Applied Biosystems 3730XL DNA sequencer. Sequenced reads were then assembled with the corresponding region of the reference genome (exported from UCSC genome browser hg19) and SNVs were confirmed using the CONSED package.^36^ All false positive SNVs identified in validation were removed from further analyzes.

The final dataset used for association analyses included 196 005 SNVs and 1 446 samples consisting of 575 cases of patients (211 cases of SCZ, 169 cases of rMDD and 195 cases of BD) and 871 controls from the LBC1936.

**Evaluation of Variant Detection and False Discovery Rates**

Using validated data from our recent sequence analysis of DISC1^1^ which included all 1446 samples that passed the quality control filters in the current study, we established a set of gold standard DISC1 variants to evaluate the quality and reliability of filtered SNV calls detected by targeted re-sequencing. In total, 1 202 gold standard DISC1 variants and 1 482 DISC1 target sequence variants were called in non-repeat regions common to both studies. Of the 1 202 gold standard DISC1 variants, 1 168 were detected by targeted resequencing estimating the sensitivity of variant detection by targeted sequencing to be approximately 97% (Supplementary Table S3). There were 314 variants specific to the targeted re-sequencing call set. Of these 314, 251 had sequence read evidence in our previous DISC1 analysis, but were not called or did not pass filtering in that study. Following Sanger validations the False Discovery Rate of variant calling by targeted sequencing was reduced to 4%. A similar FDR was achieved by randomly selecting 96 variants for Sanger validation across the entire Interactome. For the purposes of downstream variant analyses, all nonsense and splice site variants were Sanger validated and, as expected, had a higher FDR rate of 11%.

As a final measure of call quality, we contrasted the minor allele frequency (MAF) spectrum of the LBC1936 control SNVs to the MAF spectrum in the European Ancestry subset of the 1000 Genomes Project (1000G_EU). Supporting the performance of our filtering measures, the MAF spectrum of control SNVs highly correlated with the MAF spectrum in the 1000G_EU (R^2^=0.982, Supplementary Figure S6), which was a significant improvement over the correlation between the unfiltered control and 1000G_EU MAF spectra (R^2^=0.926).

**Rare Variant Burden Analysis**

We carried out general burden test (BURDEN) and sequence kernel association test (SKAT) implemented in the R package ‘SKAT’ to assess the burden of rare variants.^37^ BURDEN test was used for the gene-wide burden analysis for the genes with multiple variants (Supplementary Table S8, S11, S14, S16). BURDEN approach collapses the minor alleles across all samples into a single variable, and compares the cumulative effects in cases vs controls within a gene to evaluate the significance of the difference. However, at the gene set level, the variants in different genes affect the phenotype in different directions and most rare variants have little effect on phenotype. SKAT^37^ test is particularly designed for solving this problem. It uses a kernel machine regression approach to aggregate the associations between variants in a gene region and a phenotypic trait. SKAT is powerful for the genes with many non-causal variants or both protective and deleterious variants. Thus, we used SKAT test to evaluate the burden of rare variants at gene set level (Supplementary Table S6, S9, S13, S15).

We used a bootstrap resampling method (n.Resampling=10 000) to calculate the p-values and estimate the adjusted *P*-values using Family Wise Error Rate (FWER) correction within each trait (SCZ or BD or rMDD) (FWER_within_) and across all traits (FWER_across_). The FWER gives the probability of having at least one false-positive result when the null hypothesis (H0) is true for all M tests at α=0.05. The adjusted *P*-value is calculated by *P*=(*m* + 1)/(*n* + 1), where *n* is the total number of resampling tests (*n*=10 000) and *m* is the number of tests with a smallest *P*-value smaller than the unadjusted *P*-value from the original data set. The FWER corrections were carried out for all burden tests (functional class and frequency) within the trait (FWER_within_ *P*) and all tests across all traits (FWER_cross_ *P*). The effect size (Beta) and standard error (SE) were computed using the ‘burdenMeta’ function implemented in the R package ‘seqMeta’ for both gene-wide and gene set levels, and the odds ratio (OR) was estimated by taking exponential function of the beta value.

Due to the relatively small sample size in this study, we evaluated the rates of singleton and rare variants in each functional mutation class using the Exact Poisson test under a Poisson distribution (the sample variance is the same as the mean in our data) in cases compared to controls in each functional mutation class (Supplementary Table S7 & S10). The unadjusted *P*-value was calculated using the ‘poisson.test’ function implemented in R. As for the burden tests, we used a bootstrapping method to determine the adjusted FWER_within_ and FWER_across_ *P*-values for each test.

The case-control burden analysis was performed for each of three diagnoses (SCZ, BD and rMDD) and all three diagnoses combined (Supplementary Table S6-S11). The quantitative trait association analyses using LBC1936 controls were performed on eight traits including five cognitive measures and three personality traits described in Supplementary information: Phenotypes (Supplementary Table S13-S16). The cognitive measures were Moray House Test at age 11 (a verbal reasoning and IQ-type test), Moray House Test at age 70, Moray House Test at age 70 adjusted for the Moray House Test score at age 11, National Adult Reading Test and General Fluid Intelligence. The personality traits were Hospital Anxiety Depression Scales - Depression, Hospital Anxiety Depression Scales - Anxiety and NEO Five-Factor Inventory - Neuroticism. Both case-control and quantitative trait burden analyzes were performed for three damaging mutation classes (Disruptive, NS_strict_ and NS_broad_) on all rare variants as well as singletons as described in Supplementary information: Variant Annotation. The quantile-quantile plots showed that most of gene-wide association tests in case-control and quantitative trait association analysis follow the expected null distribution (Supplementary Figures S7 & S8).

**GO Enrichment Analysis**

Gene ontology (GO) enrichment analyses were performed using GoRilla^38^ against a background list of known protein coding genes.^39,40^ Comparison of enriched GO terms was performed using GOView.^41^ The Interactome shows significant enrichment for genes involved in cytoskeletal binding and the centrosome, particularly microtubule organization and the G2/M transition of mitotic cell cycle (Supplementary Tables S17). In contrast, the genes in the Regulome are enriched for synaptic transmission and peripheral nervous system development, particularly extracellular-glutamate-gated ion channel activity (Supplementary Tables S18). Comparison of the GO terms associated with both the DISC1 Interactome and Regulome reveals largely independent GO term associations with a very limited set of intersecting terms focused on negative regulation of cellular process, protein binding, and cell projections (Supplementary Figures S9-S11).

**TSNAXIP1**

*Translin-associated factor X interacting protein 1 (TSNAXIP1)* is highly expressed in the testis and pituitary, but also shows low levels of expression in multiple brain regions including the amygdala, caudate, frontal cortex, hippocampus, hypothalamus and nucleus accumbens (GTEx).^42^ TSNAXIP1 colocalises with TRAX showing a perinuclear localisation in non-neuronal cells.^43^ However, TRAX is known to undergo translocation to dendritic puncta in stimulated neurons and forms a Translin/TRAX complex that mediate dendritic trafficking of RNAs, including BDNF mRNA,^44^ a process thought to play a critical role in synaptic plasticity.^45^ TSNAXIP1 interacts directly with Translin-Associated Factor X (*TSNAX*)[,](#h.23ckvvd)^43^ which is located immediately upstream of *DISC1* and shows evidence for intergenic splicing with *DISC1*[.](#h.ihv636)^46^ This same upstream region has been reported to contain i) a risk haplotype for SCZ in Finnish populations.^47,48^, ii) a mediator of age of onset in depression,^49^ iii) a genetic association with depression and a regulator of DISC1 expression^50^ and iv) a binding domain for the transcriptional regulator FOXP2.^51^ The Translin/TRAX complex has recently been identified as an RNase targeting microRNAs, its activity is particularly important in the context of Dicer-deficiency.^52,53^ The RNase activity of the complex is thought to affect a subset of microRNAs and may down regulate expression of genes in the brain including neurotransmitter receptors (Glutamate receptor α3, GABA-A receptor α1) and vesicle sorting and transport proteins (Synaptobrevin-like protein, MAP-2).^54^ However, it is not known if TSNAXIP1 has any role in this complex. Further, whether DISC1 expression acts directly (and possibly reciprocally as part of a feedback loop) on TSNAXIP1 and/or indirectly via TSNAX expression remains to be determined. The association of TSNAXIP1 with SCZ may be particularly relevant given the hypothesized link between dysregulation of the microRNA machinery and neuropsychiatric disorders such as BD and SCZ^55–57^ and the regulation of Dicer expression by pathways such as MAPK and GSK-3/β-catenin.^58,59^ As the expression of translin and trax is enriched in brain where it is predominantly expressed in neurons,^56^ further studies are warranted to assess the role of this complex in regulating microRNA mediated silencing in neurons and whether inhibition of this complex may have therapeutic potential.

**URLS**

Picard: [http://picard.sourceforge.net](http://picard.sourceforge.net/)

UCSC Hg18: <http://genome.ucsc.edu/>

PLINK 1.90 beta: <https://www.cog-genomics.org/plink2>

ANNOVAR: <http://www.openbioinformatics.org/annovar/>

1000 Genomes Project: <http://www.1000genomes.org/>

Primer3: [http://primer3.sourceforge.net](http://primer3.sourceforge.net/)

SKAT: <https://cran.r-project.org/web/packages/SKAT/>

seqMeta: <http://cran.r-project.org/web/packages/seqMeta/>

**Supplementary References**

1 Thomson PA, Parla JS, McRae AF, Kramer M, Ramakrishnan K, Yao J *et al.* 708 Common and 2010 rare DISC1 locus variants identified in 1542 subjects: analysis for association with psychiatric disorder and cognitive traits. *Mol Psychiatry* 2014; **19**: 668–675.

2 Blackwood DH, Fordyce A, Walker MT, St Clair DM, Porteous DJ, Muir WJ. Schizophrenia and affective disorders--cosegregation with a translocation at chromosome 1q42 that directly disrupts brain-expressed genes: clinical and P300 findings in a family. *Am J Hum Genet* 2001; **69**: 428–433.

3 Deary IJ, Gow AJ, Taylor MD, Corley J, Brett C, Wilson V *et al.* The Lothian Birth Cohort 1936: a study to examine influences on cognitive ageing from age 11 to age 70 and beyond. *BMC Geriatr* 2007; **7**: 28.

4 Deary IJ, Yang J, Davies G, Harris SE, Tenesa A, Liewald D *et al.* Genetic contributions to stability and change in intelligence from childhood to old age. *Nature* 2012; **482**: 212–215.

5 Deary IJ, Gow AJ, Pattie A, Starr JM. Cohort profile: The lothian birth cohorts of 1921 and 1936. *Int J Epidemiol* 2012; **41**: 1576–1584.

6 Allen Frances, Harold Alan Pincus, Michael B. First et al. *Diagnostic and statistical manual of mental disorders (4th ed.)*. American Psychiatric Association, 1994.

7 Endicott J, Spitzer RL. A diagnostic interview: The Schedule for Affective Disorders and Schizophrenia. *Arch Gen Psychiatry* 1978; **35**: 837–844.

8 Gow AJ, Corley J, Starr JM, Deary IJ. Reverse causation in activity-cognitive ability associations: The Lothian Birth Cohort 1936. *Psychol Aging* 2012; **27**: 250–255.

9 Wechsler D. *WAIS‐III administration and scoring manual*. The Psychological Corporation, 1997.

10 Luciano M, Gow AJ, Harris SE, Hayward C, Allerhand M, Starr JM *et al.* Cognitive ability at age 11 and 70 years, information processing speed, and APOE variation: the Lothian Birth Cohort 1936 study. *Psychol Aging* 2009; **24**: 129–38.

11 Lacks S, Neuberger M. Membrane location of a deoxyribonuclease implicated in the genetic transformation of Diplococcus pneumoniae. *J Bacteriol* 1975; **124**: 1321–9.

12 Zigmond a S, Snaith RP. The hospital anxiety and depression scale. *Acta Psychiatr Scand* 1983; **67**: 361–370.

13 Costa, & McCrae RR. *Neo PI-R professional manual*. Odessa, FL: Psychological Assessment Resources, 1992.

14 Orchard S, Ammari M, Aranda B, Breuza L, Briganti L, Broackes-Carter F *et al.* The MIntAct project--IntAct as a common curation platform for 11 molecular interaction databases. *Nucleic Acids Res* 2014; **42**: D358-63.

15 Calderone A, Castagnoli L, Cesareni G. mentha: a resource for browsing integrated protein-interaction networks. *Nat Methods* 2013; **10**: 690–691.

16 Purcell SM, Wray NR, Stone JL, Visscher PM, O’Donovan MC, Sullivan PF *et al.* Common polygenic variation contributes to risk of schizophrenia and bipolar disorder. *Nature* 2009; **460**: 748–752.

17 Ferreira MAR, Donovan MCO, Meng YA, Jones IR, Ruderfer DM, Jones L *et al.* Collaborative genome-wide association analysis supports a role for ANK3 and CACNA1C in bipolar disorder. *Nat Genet* 2008; **40**: 1056–1058.

18 The International Schizophrenia Consortium, International T, Consortium S. Rare chromosomal deletions and duplications increase risk of schizophrenia. *Nature* 2008; **455**: 237–41.

19 Brown SM, Clapcote SJ, Millar JK, Torrance HS, Anderson SM, Walker R *et al.* Synaptic modulators Nrxn1 and Nrxn3 are disregulated in a Disc1 mouse model of schizophrenia. *Mol Psychiatry*; **16**: 585–587.

20 Hennah W, Porteous D. The DISC1 pathway modulates expression of neurodevelopmental, synaptogenic and sensory perception genes. *PLoS One* 2009; **4**: e4906.

21 Siepel A, Bejerano G, Pedersen JS, Hinrichs AS, Hou M, Rosenbloom K *et al.* Evolutionarily conserved elements in vertebrate, insect, worm, and yeast genomes. *Genome Res* 2005; **15**: 1034–1050.

22 Li H, Durbin R. Fast and accurate short read alignment with Burrows-Wheeler transform. *Bioinformatics* 2009; **25**: 1754–1760.

23 Li H, Handsaker B, Wysoker A, Fennell T, Ruan J, Homer N *et al.* The Sequence Alignment/Map format and SAMtools. *Bioinformatics* 2009; **25**: 2078–2079.

24 Barnett DW, Garrison EK, Quinlan AR, Stromberg MP, Marth GT. BamTools: a C++ API and toolkit for analyzing and managing BAM files. *Bioinformatics* 2011; **27**: 1691–1692.

25 McKenna A, Hanna M, Banks E, Sivachenko A, Cibulskis K, Kernytsky A *et al.* The Genome Analysis Toolkit: a MapReduce framework for analyzing next-generation DNA sequencing data. *Genome Res* 2010; **20**: 1297–1303.

26 Danecek P, Auton A, Abecasis G, Albers CA, Banks E, DePristo MA *et al.* The variant call format and VCFtools. *Bioinformatics* 2011; **27**: 2156–2158.

27 Purcell S, Neale B, Todd-Brown K, Thomas L, Ferreira MA, Bender D *et al.* PLINK: a tool set for whole-genome association and population-based linkage analyses. *Am J Hum Genet* 2007; **81**: 559–575.

28 Anderson CA, Pettersson FH, Clarke GM, Cardon LR, Morris AP, Zondervan KT. Data quality control in genetic case-control association studies. *Nat Protoc* 2010; **5**: 1564–73.

29 Wang K, Li M, Hakonarson H. ANNOVAR: functional annotation of genetic variants from high-throughput sequencing data. *Nucleic Acids Res* 2010; **38**: e164.

30 Ng PC, Henikoff S. SIFT: Predicting amino acid changes that affect protein function. *Nucleic Acids Res* 2003; **31**: 3812–3814.

31 Adzhubei IA, Schmidt S, Peshkin L, Ramensky VE, Gerasimova A, Bork P *et al.* A method and server for predicting damaging missense mutations. *Nat Methods* 2010; **7**: 248–249.

32 Chun S, Fay JC. Identification of deleterious mutations within three human genomes. *Genome Res* 2009; **19**: 1553–1561.

33 Schwarz JM, Rodelsperger C, Schuelke M, Seelow D. MutationTaster evaluates disease-causing potential of sequence alterations. *Nat Methods* 2010; **7**: 575–576.

34 Purcell SM, Moran JL, Fromer M, Ruderfer D, Solovieff N, Roussos P *et al.* A polygenic burden of rare disruptive mutations in schizophrenia. *Nature* 2014; **506**: 185–190.

35 McCarthy SE, Gillis J, Kramer M, Lihm J, Yoon S, Berstein Y *et al.* De novo mutations in schizophrenia implicate chromatin remodeling and support a genetic overlap with autism and intellectual disability. *Mol Psychiatry* 2014; **19**: 652–8.

36 Gordon D, Abajian C, Green P. Consed: a graphical tool for sequence finishing. *Genome Res* 1998; **8**: 195–202.

37 Wu MC, Lee S, Cai T, Li Y, Boehnke M, Lin X. Rare-variant association testing for sequencing data with the sequence kernel association test. *Am J Hum Genet* 2011; **89**: 82–93.

38 Eden E, Navon R, Steinfeld I, Lipson D, Yakhini Z. GOrilla: a tool for discovery and visualization of enriched GO terms in ranked gene lists. *BMC Bioinformatics* 2009; **10**: 48.

39 Gray KA, Yates B, Seal RL, Wright MW, Bruford EA. Genenames.org: The HGNC resources in 2015. *Nucleic Acids Res* 2015; **43**: D1079–D1085.

40 Gray KA, Seal RL, Tweedie S, Wright MW, Bruford EA. A review of the new HGNC gene family resource. *Hum Genomics* 2016; **10**: 6.

41 Zhang B, Kirov S, Snoddy J. WebGestalt: An integrated system for exploring gene sets in various biological contexts. *Nucleic Acids Res* 2005; **33**: 741–748.

42 GTEx Consortium J, Thomas J, Salvatore M, Phillips R, Lo E, Shad S *et al.* The Genotype-Tissue Expression (GTEx) project. *Nat Genet* 2013; **45**: 580–5.

43 Bray JD, Chennathukuzhi VM, Hecht NB. Identification and characterization of cDNAs encoding four novel proteins that interact with translin associated factor-X. *Genomics* 2002; **79**: 799–808.

44 Wu Y-C, Williamson R, Li Z, Vicario A, Xu J, Kasai M *et al.* Dendritic trafficking of brain-derived neurotrophic factor mRNA: regulation by translin-dependent and -independent mechanisms. *J Neurochem* 2011; **116**: 1112–21.

45 Li Z, Wu Y, Baraban JM. The Translin/Trax RNA binding complex: clues to function in the nervous system. *Biochim Biophys Acta* 2008; **1779**: 479–85.

46 Millar JK, Christie S, Semple CA, Porteous DJ. Chromosomal location and genomic structure of the human translin-associated factor X gene (TRAX; TSNAX) revealed by intergenic splicing to DISC1, a gene disrupted by a translocation segregating with schizophrenia. *Genomics* 2000; **67**: 69–77.

47 Palo OM, Antila M, Silander K, Hennah W, Kilpinen H, Soronen P *et al.* Association of distinct allelic haplotypes of DISC1 with psychotic and bipolar spectrum disorders and with underlying cognitive impairments. *Hum Mol Genet* 2007; **16**: 2517–2528.

48 Cannon TD, Hennah W, van Erp TGM, Thompson PM, Lonnqvist J, Huttunen M *et al.* Association of DISC1/TRAX haplotypes with schizophrenia, reduced prefrontal gray matter, and impaired short- and long-term memory. *Arch Gen Psychiatry* 2005; **62**: 1205–13.

49 Thomson PA, MacIntyre DJ, Hamilton G, Dominiczak A, Smith BH, Morris A *et al.* Association of DISC1 variants with age of onset in a population-based sample of recurrent major depression. *Mol Psychiatry* 2013; **18**: 745–7.

50 Carless MA, Glahn DC, Johnson MP, Curran JE, Bozaoglu K, Dyer TD *et al.* Impact of DISC1 variation on neuroanatomical and neurocognitive phenotypes. *Mol Psychiatry* 2011; **16**: 1063,1096-1104.

51 Walker RM, Hill AE, Newman AC, Hamilton G, Torrance HS, Anderson SM *et al.* The DISC1 promoter: Characterization and regulation by FOXP2. *Hum Mol Genet* 2012; **21**: 2862–2872.

52 Asada K, Canestrari E, Fu X, Li Z, Makowski E, Wu YC *et al.* Rescuing dicer Defects via Inhibition of an Anti-Dicing Nuclease. *Cell Rep* 2014; **9**: 1471–1481.

53 Fu X, Shah A, Baraban JM. Rapid reversal of translational silencing: Emerging role of microRNA degradation pathways in neuronal plasticity. *Neurobiol Learn Mem* 2016; **133**: 225–32.

54 Chennathukuzhi V, Stein JM, Abel T, Donlon S, Yang S, Miller JP *et al.* Mice deficient for testis-brain RNA-binding protein exhibit a coordinate loss of TRAX, reduced fertility, altered gene expression in the brain, and behavioral changes. *Mol Cell Biol* 2003; **23**: 6419–6434.

55 Geaghan M, Cairns MJ. MicroRNA and Posttranscriptional Dysregulation in Psychiatry. *Biol Psychiatry* 2014; **78**: 231–239.

56 Finkenstadt PM, Kang WS, Jeon M, Taira E, Tang W, Baraban JM. Somatodendritic localization of translin, a component of the Translin/Trax RNA binding complex. *J Neurochem* 2000; **75**: 1754–1762.

57 Sun E, Shi Y. MicroRNAs: Small molecules with big roles in neurodevelopment and diseases. *Exp Neurol* 2015; **268**: 46–53.

58 Huang YWA, Ruiz CR, Eyler ECH, Lin K, Meffert MK. Dual regulation of miRNA biogenesis generates target specificity in neurotrophin-induced protein synthesis. *Cell* 2012; **148**: 933–946.

59 Dias C, Feng J, Sun H, Shao NY, Mazei-Robison MS, Damez-Werno D *et al.* β-catenin mediates stress resilience through Dicer1/microRNA regulation. *Nature* 2014; **516**: 51–5.

**Supplementary Tables**

**Supplementary Table S1: DISC1 Interactome and Regulome Gene List**

The full list of 213 gene symbols and coordinates (hg18 & hg19).

See: “S1.GeneList” sheet in supplementary tables.xlsx

**Supplementary Table S2: Sequencing Summary Statistics**

Averages for Mean Target Coverage and Percentage of the Target Bases at 2x, 10x, 20x and 30x are shown for all samples (ALL) as well as individually for schizophrenia (SCZ), recurrent major depressive disorder (rMDD), bipolar (BD), and the Lothian Birth Cohort of 1936 (LBC1936) samples. The samples with 80% of the targets at ≥20x read depth were used for further analysis. The case control quality control filters (Supplementary Figure S2) were applied to generate the final data set.

|  | **ALL** | **SCZ** | **rMDD** | **BD** | **LBC1936** |
| --- | --- | --- | --- | --- | --- |
| All Sequenced Sample | | | | | |
| Total Number | 1543 | 241 | 192 | 221 | 889 |
| Mean Target Coverage | 115 | 105 | 107 | 111 | 120 |
| Percentage of Target Bases at ≥2x | 96.96% | 96.43% | 96.55% | 96.90% | 97.21% |
| Percentage of Target Bases at ≥10x | 94.21% | 92.82% | 92.51% | 93.56% | 95.11% |
| Percentage of Target Bases at ≥20x | 90.11% | 87.79% | 87.00% | 88.71% | 91.76% |
| Percentage of Target Bases at ≥30x | 85.23% | 82.09% | 81.02% | 83.05% | 87.53% |
| Samples with 80% of the Targets at ≥20x | | | | | |
| Total Number | 1464 | 217 | 173 | 199 | 875 |
| Mean Target Coverage | 118 | 111 | 113 | 117 | 121 |
| Percentage of Target Bases at ≥2x | 97.15% | 97.08% | 97.03% | 97.03% | 97.22% |
| Percentage of Target Bases at ≥10x | 94.84% | 94.42% | 94.19% | 94.23% | 95.21% |
| Percentage of Target Bases at ≥20x | 91.27% | 90.35% | 89.91% | 90.17% | 92.02% |
| Percentage of Target Bases at ≥30x | 86.84% | 85.35% | 84.74% | 85.28% | 87.98% |
| Samples in the Final Data Set | | | | | |
| Total Number | 1446 | 211 | 169 | 195 | 871 |
| Mean Target Coverage | 118 | 111 | 113 | 117 | 121 |
| Percentage of Target Bases at ≥2x | 97.15% | 97.08% | 97.04% | 97.04% | 97.22% |
| Percentage of Target Bases at ≥10x | 94.85% | 94.42% | 94.22% | 94.28% | 95.21% |
| Percentage of Target Bases at ≥20x | 91.30% | 90.35% | 89.95% | 90.25% | 92.03% |
| Percentage of Target Bases at ≥30x | 86.88% | 85.35% | 84.76% | 85.40% | 87.99% |

**Supplementary Table S3: Variant Summary Statistics**

| **Class** | |  | **# SNV** | **% Rare**  **(MAF<1%)** | **% Singleton** |
| --- | --- | --- | --- | --- | --- |
| All Identified SNVs | | | 196080 | 78% | 50% |
|  | Common (MAF ≥ 1%) | | 42789 |  |  |
|  | Rare (MAF<1%) | | 153291 |  |  |
|  |  | Singleton | 97786 |  |  |
|  |  |  |  |  |  |
| Reported in Public Databases | | |  |  |  |
|  | 1000G_EU^a^ | | 77593 |  |  |
|  | dbSNP144 | | 124268 |  |  |
|  |  |  |  |  |  |
| Functional Anotations | | |  |  |  |
|  | Intronic | | 169905 | 78% | 50% |
|  | 5'/3' UTR | | 5410 | 84% | 53% |
|  | Exonic | | 4523 | 86% | 57% |
|  |  | Silent | 1893 | 79% | 51% |
|  |  | Missense | 2569 | 91% | 62% |
|  |  | Nonsense | 41 | 100% | 71% |
|  |  | Unknown^b^ | 20 | 90% | 65% |
|  | Splice Site | | 24 | 92% | 75% |
|  | Damaging Mutations^c^ | |  |  |  |
|  |  | Disruptive | 65 | 97% | 72% |
|  |  | NS_strict_ | 374 | 98% | 73% |
|  |  | NS_broad_ | 2057 | 94% | 66% |

^a^Based on ANNOVAR filter for 1000 Genomes Project (2015 Aug) European subset annotations.

^b^Unknown refers to exonic variants in genes with incomplete or unavailable ORF information. These variants were not included in the analysis.

^c^Disruptive, nonsense and splice site variants; NS_strict_, Non-synonymous strict damaging mutations were defined as disruptive plus missense variants predicted as damaging by all five algorithms (PolyPhen2 HumDiv and HumVar, SIFT, LRT and MutationTaster); NS_broad_, Non-synonymous broad damaging mutations were defined as disruptive plus missense variants predicted as damaging by at least one algorithm above.

**Supplementary Table S4: Sensitivity and Specificity of DISC1 Variant Discovery by Capture Sequencing**

The DISC1 Locus (DL) variants were obtained from the recent DISC1 locus sequence analysis[^1^](#_ENREF_1) and validated using Sanger sequencing. The DISC1 Interactome (DI) variants identified in the present study were filtered by case control quality control filters (Supplementary Figure S2). Both DL and DI variants were called in *DISC1* non-repeat regions common to both studies.

|  | **DISC1 Locus  (DL)** | | **Total** |
| --- | --- | --- | --- |
|  | **Validated Variants** | |  |
| **DISC1 Interactome (DI)** | **DI in DL** | **DI not in DL** | 1482 |
|  | 1168 | 314 |  |
| **Filtered Variants** | **DL not in DI** | **Not in DL or DI** | 136173 |
|  | 34 | 136139 |  |
| **Total** | 1202 | 136453 | 137655 |

Supplementary Table S5: Summary of Validated Disruptive Variants

| **Gene** | **Chr** | **Pos (hg18)** | **Ref base** | **Alt base** | **dbSNP144** | **1000G-Eur Frequency** | **Case MAF** | **Control MAF** | **SCZ^a^** | **rMDD^a^** | **BP^a^** | **CTL^a^** |
| --- | --- | --- | --- | --- | --- | --- | --- | --- | --- | --- | --- | --- |
| **Nonsense** | | | | | | | | | | | | |
| AP4B1 | 1 | 114246002 | G | A | NA | NA | 0.0009 | 0.0006 | 0/0/211 | 0/0/169 | 0/1/194 | 0/1/870 |
| DISC1 | 1 | 229924937 | G | A | rs201177890 | NA | 0.0000 | 0.0006 | 0/0/211 | 0/0/169 | 0/0/195 | 0/1/870 |
| DISC1 | 1 | 230211206 | C | T | rs190975963 | NA | 0.0009 | 0.0006 | 0/0/211 | 0/1/168 | 0/0/195 | 0/1/870 |
| NRXN1 | 2 | 50704217 | G | C | NA | NA | 0.0009 | 0.0000 | 0/1/210 | 0/0/169 | 0/0/195 | 0/0/871 |
| NRXN1 | 2 | 51108309 | T | A | NA | NA | 0.0009 | 0.0000 | 0/1/210 | 0/0/169 | 0/0/195 | 0/0/871 |
| DCTN1 | 2 | 74454963 | G | A | NA | NA | 0.0000 | 0.0006 | 0/0/211 | 0/0/169 | 0/0/195 | 0/1/870 |
| SH3BP5 | 3 | 15286253 | G | A | NA | NA | 0.0009 | 0.0000 | 0/0/211 | 0/1/166 | 0/0/193 | 0/0/871 |
| KALRN | 3 | 125786386 | C | T | rs56407180 | 0.0010 | 0.0009 | 0.0023 | 0/0/211 | 0/0/169 | 0/1/194 | 0/4/867 |
| NEK1 | 4 | 170582394 | G | C | rs199947197 | NA | 0.0000 | 0.0006 | 0/0/211 | 0/0/169 | 0/0/195 | 0/1/870 |
| DPYSL3 | 5 | 146775486 | G | A | NA | NA | 0.0009 | 0.0000 | 0/0/211 | 0/1/168 | 0/0/195 | 0/0/871 |
| DTNBP1 | 6 | 15632694 | G | A | rs144524387 | NA | 0.0009 | 0.0006 | 0/1/210 | 0/0/169 | 0/0/195 | 0/1/870 |
| DTNBP1 | 6 | 15771061 | C | A | NA | NA | 0.0000 | 0.0006 | 0/0/211 | 0/0/168 | 0/0/192 | 0/1/869 |
| DST | 6 | 56593325 | G | A | rs577972555 | NA | 0.0009 | 0.0000 | 0/0/211 | 0/0/169 | 0/1/194 | 0/0/871 |
| SYNE1 | 6 | 152587381 | G | A | rs778445117 | NA | 0.0000 | 0.0006 | 0/0/211 | 0/0/169 | 0/0/195 | 0/1/870 |
| NUDT1 | 7 | 2256028 | G | T | rs370549369 | NA | 0.0010 | 0.0000 | 0/0/196 | 0/0/148 | 0/1/166 | 0/0/839 |
| MCPH1 | 8 | 6289896 | T | A | rs377204886 | NA | 0.0009 | 0.0000 | 0/0/211 | 0/0/169 | 0/1/194 | 0/0/871 |
| PCM1 | 8 | 17882493 | G | T | rs148806955 | 0.0050 | 0.0035 | 0.0029 | 0/3/208 | 0/1/168 | 0/0/195 | 0/5/866 |
| DMRT2 | 9 | 1046377 | C | T | NA | NA | 0.0009 | 0.0000 | 0/0/211 | 0/1/168 | 0/0/195 | 0/0/871 |
| KCNQ1 | 11 | 2746687 | C | T | rs17215500 | NA | 0.0018 | 0.0000 | 0/0/205 | 0/0/157 | 0/2/178 | 0/0/859 |
| CEP290 | 12 | 86995171 | C | A | rs137852832 | NA | 0.0000 | 0.0006 | 0/0/211 | 0/0/169 | 0/0/195 | 0/1/870 |
| CEP290 | 12 | 87001844 | T | A | rs137852834 | NA | 0.0009 | 0.0017 | 0/0/211 | 0/0/169 | 0/1/194 | 0/3/868 |
| CEP290 | 12 | 87032396 | G | A | rs386834152 | NA | 0.0000 | 0.0011 | 0/0/211 | 0/0/169 | 0/0/195 | 0/2/869 |
| CEP290 | 12 | 87049117 | G | A | rs757641323 | NA | 0.0000 | 0.0011 | 0/0/211 | 0/0/169 | 0/0/195 | 0/2/869 |
| APPL2 | 12 | 104113408 | C | T | NA | NA | 0.0017 | 0.0006 | 0/2/209 | 0/0/169 | 0/0/195 | 0/1/870 |
| APPL2 | 12 | 104115860 | T | A | NA | NA | 0.0000 | 0.0006 | 0/0/211 | 0/0/169 | 0/0/195 | 0/1/870 |
| MAP1A | 15 | 41609340 | C | T | NA | NA | 0.0000 | 0.0006 | 0/0/210 | 0/0/169 | 0/0/194 | 0/1/870 |
| CHRNA5 | 15 | 76666084 | C | T | NA | NA | 0.0009 | 0.0000 | 0/1/210 | 0/0/169 | 0/0/195 | 0/0/871 |
| SV2B | 15 | 89602698 | G | A | NA | NA | 0.0000 | 0.0006 | 0/0/211 | 0/0/169 | 0/0/195 | 0/1/870 |
| EEF2K | 16 | 22181932 | G | T | rs139935693 | NA | 0.0009 | 0.0011 | 0/1/210 | 0/0/169 | 0/0/195 | 0/2/869 |
| PRKCB | 16 | 24110023 | G | T | NA | NA | 0.0009 | 0.0000 | 0/1/210 | 0/0/169 | 0/0/195 | 0/0/871 |
| TSNAXIP1 | 16 | 66405794 | C | T | rs146214814 | 0.0030 | 0.0054 | 0.0041 | 0/6/200 | 0/0/163 | 0/0/189 | 0/7/854 |
| PCNT | 21 | 46656167 | G | T | NA | NA | 0.0000 | 0.0006 | 0/0/176 | 0/0/137 | 0/0/164 | 0/1/845 |
| PCNT | 21 | 46688165 | C | T | NA | NA | 0.0009 | 0.0000 | 0/1/210 | 0/0/169 | 0/0/195 | 0/0/871 |
| TRIOBP | 22 | 36449548 | C | T | rs118204026 | NA | 0.0000 | 0.0006 | 0/0/211 | 0/0/163 | 0/0/188 | 0/1/870 |
| **Splice site** | | | | | | | | | | | | |
| BLZF1 | 1 | 167604205 | G | A | rs187961364 | 0.0030 | 0.0113 | 0.0138 | 0/5/206 | 0/3/166 | 0/5/190 | 0/24/847 |
| LPIN1 | 2 | 11771390 | G | T | NA | NA | 0.0009 | 0.0000 | 0/0/211 | 0/1/168 | 0/0/195 | 0/0/871 |
| SYN2 | 3 | 12186408 | T | G | rs773718233 | NA | 0.0000 | 0.0006 | 0/0/211 | 0/0/169 | 0/0/195 | 0/1/870 |
| SNAP91 | 6 | 84347024 | T | C | NA | NA | 0.0009 | 0.0006 | 0/0/211 | 0/1/168 | 0/0/195 | 0/1/870 |
| SYNE1 | 6 | 152991092 | C | A | NA | NA | 0.0009 | 0.0000 | 0/0/211 | 0/1/168 | 0/0/195 | 0/0/871 |
| PCM1 | 8 | 17824978 | G | A | rs375662391 | NA | 0.0009 | 0.0000 | 0/1/209 | 0/0/169 | 0/0/195 | 0/0/870 |
| PCM1 | 8 | 17826500 | A | T | NA | NA | 0.0009 | 0.0000 | 0/1/210 | 0/0/169 | 0/0/195 | 0/0/871 |
| PCM1 | 8 | 17826570 | G | T | rs747885172 | NA | 0.0009 | 0.0000 | 0/0/211 | 0/0/169 | 0/1/194 | 0/0/871 |
| RAD21 | 8 | 117948182 | C | T | rs16889042 | 0.0040 | 0.0009 | 0.0023 | 0/0/211 | 0/1/168 | 0/0/195 | 0/4/867 |
| SLC1A2 | 11 | 35300683 | C | T | rs56205617 | 0.0338 | 0.0339 | 0.0276 | 0/21/190 | 0/5/164 | 0/13/182 | 0/48/823 |
| DIXDC1 | 11 | 111358273 | G | A | NA | NA | 0.0000 | 0.0006 | 0/0/211 | 0/0/169 | 0/0/195 | 0/1/870 |
| DIXDC1 | 11 | 111371043 | G | A | rs200500225 | NA | 0.0000 | 0.0006 | 0/0/211 | 0/0/169 | 0/0/195 | 0/1/870 |
| CEP290 | 12 | 87038904 | C | A | NA | NA | 0.0000 | 0.0006 | 0/0/211 | 0/0/169 | 0/0/195 | 0/1/870 |
| APPL2 | 12 | 104147133 | T | C | NA | NA | 0.0000 | 0.0006 | 0/0/211 | 0/0/169 | 0/0/195 | 0/1/870 |
| EEF2K | 16 | 22169514 | G | A | rs200670923 | NA | 0.0009 | 0.0000 | 0/1/210 | 0/0/169 | 0/0/195 | 0/0/871 |
| TSNAXIP1 | 16 | 66419156 | G | C | NA | NA | 0.0009 | 0.0000 | 0/0/210 | 0/1/163 | 0/0/189 | 0/0/868 |
| STAT5A | 17 | 37705293 | A | T | NA | NA | 0.0000 | 0.0006 | 0/0/211 | 0/0/169 | 0/0/195 | 0/1/870 |
| CDK5RAP3 | 17 | 43413801 | G | C | rs184760380 | 0.0010 | 0.0000 | 0.0006 | 0/0/211 | 0/0/169 | 0/0/195 | 0/1/870 |
| PCNT | 21 | 46642354 | A | G | rs760664460 | NA | 0.0009 | 0.0006 | 0/0/210 | 0/1/167 | 0/0/195 | 0/1/870 |
| BCR | 22 | 21986153 | A | C | NA | NA | 0.0009 | 0.0000 | 0/1/209 | 0/0/164 | 0/0/189 | 0/0/870 |

^a^Genotype counts are shown for Schizophrenia (SCZ), bipolar (BD), recurrent major depressive disorder (rMDD) cases and the Lothian Birth Cohort of 1936 controls (CTL).

Supplementary Table S6. Gene Set Burden Analysis of Rare Functional Variants in the DISC1 Interactome for Case-Control Traits

| **Trait^a^** | **Mutation Class^b^** | **MAFbin** | **N.SNP.Test** | **Unadjusted *P*** | **FWER_within_ *P*** | **FWER_cross_ *P*** | **OR** | **SE** |
| --- | --- | --- | --- | --- | --- | --- | --- | --- |
| SCZ | Disruptive | Singleton | 11 | 0.8085 | 0.9992 | 1.0000 | 0.9021 | 0.1201 |
| SCZ | Disruptive | Rare | 19 | 0.8270 | 0.9996 | 1.0000 | 0.8487 | 0.0746 |
| SCZ | NS_strict_ | Singleton | 94 | 0.7180 | 0.9915 | 1.0000 | 0.9768 | 0.0405 |
| SCZ | NS_strict_ | Rare | 147 | 0.9091 | 0.9998 | 1.0000 | 0.9818 | 0.0242 |
| SCZ | NS_broad_ | Singleton | 451 | 0.2572 | 0.7552 | 0.9962 | 1.0124 | 0.0178 |
| SCZ | NS_broad_ | Rare | 721 | 0.7876 | 0.9967 | 1.0000 | 0.9959 | 0.0095 |
| rMDD | Disruptive | Singleton | 13 | 0.2484 | 0.7102 | 0.9952 | 1.0736 | 0.1030 |
| rMDD | Disruptive | Rare | 21 | 0.4635 | 0.8953 | 1.0000 | 1.0154 | 0.0644 |
| rMDD | NS_strict_ | Singleton | 97 | 0.1955 | 0.5473 | 0.9747 | 1.0344 | 0.0369 |
| rMDD | NS_strict_ | Rare | 150 | 0.3592 | 0.8258 | 0.9997 | 1.0110 | 0.0225 |
| rMDD | NS_broad_ | Singleton | 441 | 0.1232 | 0.4167 | 0.9012 | 1.0262 | 0.0174 |
| rMDD | NS_broad_ | Rare | 708 | 0.2849 | 0.7605 | 0.9979 | 1.0002 | 0.0090 |
| BD | Disruptive | Singleton | 11 | 0.7858 | 0.9986 | 1.0000 | 0.9130 | 0.1172 |
| BD | Disruptive | Rare | 20 | 0.3831 | 0.8461 | 0.9997 | 0.9946 | 0.0674 |
| BD | NS_strict_ | Singleton | 91 | 0.8326 | 0.9994 | 1.0000 | 0.9639 | 0.0396 |
| BD | NS_strict_ | Rare | 144 | 0.9167 | 1.0000 | 1.0000 | 0.9605 | 0.0239 |
| BD | NS_broad_ | Singleton | 436 | 0.5918 | 0.9723 | 1.0000 | 0.9971 | 0.0181 |
| BD | NS_broad_ | Rare | 700 | 0.4302 | 0.8832 | 0.9998 | 0.9910 | 0.0094 |
| Combined | Disruptive | Singleton | 15 | 0.7145 | 0.9968 | 1.0000 | 0.9396 | 0.1271 |
| Combined | Disruptive | Rare | 24 | 0.6307 | 0.9881 | 1.0000 | 0.9219 | 0.0776 |
| Combined | NS_strict_ | Singleton | 126 | 0.6553 | 0.9905 | 1.0000 | 0.9857 | 0.0431 |
| Combined | NS_strict_ | Rare | 181 | 0.9209 | 1.0000 | 1.0000 | 0.9740 | 0.0259 |
| Combined | NS_broad_ | Singleton | 612 | 0.2355 | 0.7185 | 0.9905 | 1.0184 | 0.0192 |
| Combined | NS_broad_ | Rare | 888 | 0.3625 | 0.8827 | 0.9997 | 0.9929 | 0.0101 |

^a^Schizophrenia (SCZ); bipolar disorder (BD); recurrent major depressive disorder (rMDD); Combined Cases (Combined).

^b^Non-synonymous strictly damaging mutations (NS_strict_); Non-synonymous broadly damaging mutations (NS_broad_).

The odds ratio (OR) and standard error (SE).

**Supplementary Table S7. Gene Set Exact Poisson Tests of Rare Functional Variants in the DISC1 Interactome for Case-Control Traits.**

| **Trait^a^** | **Mutation Class^b^** | **MAFbin** | **Unadjusted *P*** | **FWER_within_ *P*** | **FWER_cross_ *P*** | **Case/Control Ratio** | **Control Rate** | **Case Rate** |
| --- | --- | --- | --- | --- | --- | --- | --- | --- |
| SCZ | Disruptive | Singleton | 0.7397 | 0.9981 | 1.0000 | 0.4128 | 0.0115 | 0.0047 |
| SCZ | Disruptive | Rare | **0.0188** | 0.1683 | 0.5666 | 0.1474 | 0.0321 | 0.0047 |
| SCZ | NS_strict_ | Singleton | 0.6440 | 0.9948 | 1.0000 | 0.8468 | 0.0896 | 0.0758 |
| SCZ | NS_strict_ | Rare | 0.4505 | 0.9504 | 1.0000 | 0.8819 | 0.2526 | 0.2227 |
| SCZ | NS_broad_ | Singleton | 0.4850 | 0.9711 | 1.0000 | 1.0723 | 0.4110 | 0.4408 |
| SCZ | NS_broad_ | Rare | 0.5994 | 0.9926 | 1.0000 | 0.9686 | 1.5511 | 1.5024 |
| rMDD | Disruptive | Singleton | 0.4508 | 0.9539 | 1.0000 | 1.5462 | 0.0115 | 0.0178 |
| rMDD | Disruptive | Rare | 0.6690 | 0.9908 | 1.0000 | 1.1044 | 0.0321 | 0.0355 |
| rMDD | NS_strict_ | Singleton | 0.3022 | 0.8348 | 0.9994 | 1.2554 | 0.0896 | 0.1124 |
| rMDD | NS_strict_ | Rare | 0.5916 | 0.9842 | 1.0000 | 1.0776 | 0.2526 | 0.2722 |
| rMDD | NS_broad_ | Singleton | 0.1051 | 0.4827 | 0.9612 | 1.1949 | 0.4110 | 0.4911 |
| rMDD | NS_broad_ | Rare | 1.0000 | 1.0000 | 1.0000 | 0.9995 | 1.5511 | 1.5503 |
| BD | Disruptive | Singleton | 0.7329 | 0.9992 | 1.0000 | 0.4467 | 0.0115 | 0.0051 |
| BD | Disruptive | Rare | 1.0000 | 1.0000 | 1.0000 | 0.9571 | 0.0321 | 0.0308 |
| BD | NS_strict_ | Singleton | 0.3379 | 0.9124 | 0.9999 | 0.7444 | 0.0896 | 0.0667 |
| BD | NS_strict_ | Rare | 0.0633 | 0.3401 | 0.8611 | 0.7309 | 0.2526 | 0.1846 |
| BD | NS_broad_ | Singleton | 0.8669 | 1.0000 | 1.0000 | 0.9732 | 0.4110 | 0.4000 |
| BD | NS_broad_ | Rare | 0.2272 | 0.7571 | 0.9971 | 0.9290 | 1.5511 | 1.4410 |
| Combined | Disruptive | Singleton | 0.6966 | 0.9994 | 1.0000 | 0.7574 | 0.0115 | 0.0087 |
| Combined | Disruptive | Rare | 0.2433 | 0.8779 | 0.9985 | 0.7033 | 0.0321 | 0.0226 |
| Combined | NS_strict_ | Singleton | 0.6761 | 0.9988 | 1.0000 | 0.9322 | 0.0896 | 0.0835 |
| Combined | NS_strict_ | Rare | 0.1843 | 0.8146 | 0.9938 | 0.8882 | 0.2526 | 0.2243 |
| Combined | NS_broad_ | Singleton | 0.2548 | 0.8975 | 0.9987 | 1.0747 | 0.4110 | 0.4417 |
| Combined | NS_broad_ | Rare | 0.2915 | 0.9118 | 0.9993 | 0.9643 | 1.5511 | 1.4957 |

^a^Schizophrenia (SCZ); bipolar disorder (BD); recurrent major depressive disorder (rMDD); Combined Cases (Combined).

^b^Non-synonymous strictly damaging mutations (NSstrict); Non-synonymous broadly damaging mutations (NSbroad).

**Bold**, P<0.05; Case/Control Ratio is calculated using Case Rate divided by Control Rate.

**Supplementary Table S8. Gene-Wide Burden Analysis of Rare Functional Variants in the DISC1 Interactome for Case-Control Traits**

See: “S8.CaseControlGeneInteractome” sheet in supplementary tables.xlsx

^a^Schizophrenia (SCZ); bipolar disorder (BD); recurrent major depressive disorder (rMDD); Combined Cases (Combined).

^b^Non-synonymous strictly damaging mutations (NSstrict); Non-synonymous broadly damaging mutations (NSbroad).

**Bold**, *P*<0.05. Tests are sorted in ascending order of the unadjusted *P*-value. The odds ratio (OR) and standard error (SE).

Supplementary Table S9. Gene Set Burden Analysis of Rare Functional Variants in the DISC1 Regulome for Case-Control Traits

| **Trait^a^** | **Mutation Class^b^** | **MAFbin** | **N.SNP.Test** | **Unadjusted *P*** | **FWER_within_ *P*** | **FWER_cross_ *P*** | **OR** | **SE** |
| --- | --- | --- | --- | --- | --- | --- | --- | --- |
| SCZ | Disruptive | Singleton | 16 | **0.0019** | **0.0069** | **0.0339** | 1.3162 | 0.0941 |
| SCZ | Disruptive | Rare | 22 | **0.0061** | **0.0228** | 0.0863 | 1.2992 | 0.0584 |
| SCZ | NS_strict_ | Singleton | 136 | **0.0314** | 0.1106 | 0.3852 | 1.0776 | 0.0335 |
| SCZ | NS_strict_ | Rare | 186 | **0.0398** | 0.1385 | 0.4694 | 1.0685 | 0.0227 |
| SCZ | NS_broad_ | Singleton | 570 | 0.1032 | 0.3424 | 0.8302 | 1.0227 | 0.0158 |
| SCZ | NS_broad_ | Rare | 883 | 0.1063 | 0.3504 | 0.8384 | 1.0081 | 0.0090 |
| rMDD | Disruptive | Singleton | 11 | 0.1580 | 0.4736 | 0.9378 | 1.1188 | 0.1119 |
| rMDD | Disruptive | Rare | 17 | 0.3287 | 0.7683 | 0.9989 | 1.0054 | 0.0684 |
| rMDD | NS_strict_ | Singleton | 122 | 0.2557 | 0.6828 | 0.9920 | 1.0259 | 0.0328 |
| rMDD | NS_strict_ | Rare | 172 | 0.1281 | 0.3933 | 0.8843 | 1.0209 | 0.0220 |
| rMDD | NS_broad_ | Singleton | 536 | 0.5029 | 0.9301 | 1.0000 | 1.0036 | 0.0152 |
| rMDD | NS_broad_ | Rare | 842 | 0.8302 | 0.9985 | 1.0000 | 0.9924 | 0.0086 |
| BD | Disruptive | Singleton | 11 | 0.2107 | 0.5821 | 0.9795 | 1.0974 | 0.1172 |
| BD | Disruptive | Rare | 17 | 0.2527 | 0.7082 | 0.9915 | 0.9266 | 0.0741 |
| BD | NS_strict_ | Singleton | 116 | 0.8479 | 0.9990 | 1.0000 | 0.9654 | 0.0353 |
| BD | NS_strict_ | Rare | 165 | 0.7288 | 0.9923 | 1.0000 | 0.9477 | 0.0235 |
| BD | NS_broad_ | Singleton | 553 | 0.2796 | 0.7375 | 0.9945 | 1.0110 | 0.0155 |
| BD | NS_broad_ | Rare | 858 | 0.2574 | 0.7172 | 0.9921 | 0.9978 | 0.0088 |
| Combined | Disruptive | Singleton | 22 | **0.0127** | **0.0461** | 0.2112 | 1.2503 | 0.1006 |
| Combined | Disruptive | Rare | 28 | 0.8971 | 1.0000 | 1.0000 | 1.1515 | 0.0666 |
| Combined | NS_strict_ | Singleton | 176 | 0.1623 | 0.5365 | 0.9492 | 1.0410 | 0.0368 |
| Combined | NS_strict_ | Rare | 227 | 0.8261 | 0.9989 | 1.0000 | 1.0228 | 0.0250 |
| Combined | NS_broad_ | Singleton | 765 | 0.1455 | 0.5003 | 0.9228 | 1.0206 | 0.0168 |
| Combined | NS_broad_ | Rare | 1084 | 0.1972 | 0.6431 | 0.9733 | 0.9995 | 0.0096 |

^a^Schizophrenia (SCZ); bipolar disorder (BD); recurrent major depressive disorder (rMDD); Combined Cases (Combined).

^b^Non-synonymous strictly damaging mutations (NS_strict_); Non-synonymous broadly damaging mutations (NS_broad_).

**Bold**, *P*<0.05. The odds ratio (OR) and standard error (SE).

Supplementary Table S10. Gene Set Exact Poisson Tests of Rare Functional Variants in the DISC1 Regulome for Case-Control Traits

| **Trait^a^** | **Mutation Class^b^** | **MAFbin** | **Unadjusted *P*** | **FWER_within_ *P*** | **FWER_cross_ *P*** | **Case/Control Ratio** | **Control Rate** | **Case Rate** |
| --- | --- | --- | --- | --- | --- | --- | --- | --- |
| SCZ | Disruptive | Singleton | **9.00E-04** | **0.0185** | 0.0965 | 4.1280 | 0.0092 | 0.0379 |
| SCZ | Disruptive | Rare | **1.68E-06** | **1.00E-04** | **0.0022** | 3.4675 | 0.0287 | 0.0995 |
| SCZ | NS_strict_ | Singleton | **0.0136** | 0.1249 | 0.5022 | 1.5428 | 0.1137 | 0.1754 |
| SCZ | NS_strict_ | Rare | **0.0013** | **0.0304** | 0.1434 | 1.4624 | 0.2560 | 0.3744 |
| SCZ | NS_broad_ | Singleton | 0.1631 | 0.6385 | 0.9892 | 1.1359 | 0.5132 | 0.5829 |
| SCZ | NS_broad_ | Rare | 0.4477 | 0.9501 | 1.0000 | 1.0408 | 1.6119 | 1.6777 |
| rMDD | Disruptive | Singleton | 0.2044 | 0.6908 | 0.9972 | 1.9327 | 0.0092 | 0.0178 |
| rMDD | Disruptive | Rare | 0.8195 | 0.9996 | 1.0000 | 1.0308 | 0.0287 | 0.0296 |
| rMDD | NS_strict_ | Singleton | 0.3605 | 0.9047 | 1.0000 | 1.1974 | 0.1137 | 0.1361 |
| rMDD | NS_strict_ | Rare | 0.2873 | 0.8662 | 0.9997 | 1.1556 | 0.2560 | 0.2959 |
| rMDD | NS_broad_ | Singleton | 0.7882 | 0.9995 | 1.0000 | 1.0262 | 0.5132 | 0.5266 |
| rMDD | NS_broad_ | Rare | 0.2496 | 0.8163 | 0.9990 | 0.9287 | 1.6119 | 1.4970 |
| BD | Disruptive | Singleton | 0.2670 | 0.8207 | 0.9993 | 1.6750 | 0.0092 | 0.0154 |
| BD | Disruptive | Rare | 0.3936 | 0.9287 | 1.0000 | 0.5360 | 0.0287 | 0.0154 |
| BD | NS_strict_ | Singleton | 0.3374 | 0.9000 | 0.9998 | 0.7670 | 0.1137 | 0.0872 |
| BD | NS_strict_ | Rare | **0.0087** | 0.0910 | 0.4004 | 0.6410 | 0.2560 | 0.1641 |
| BD | NS_broad_ | Singleton | 0.5483 | 0.9842 | 1.0000 | 1.0592 | 0.5132 | 0.5436 |
| BD | NS_broad_ | Rare | 0.5165 | 0.9826 | 1.0000 | 0.9608 | 1.6119 | 1.5487 |
| Combined | Disruptive | Singleton | **0.0012** | 0.0616 | 0.1211 | 2.6509 | 0.0092 | 0.0243 |
| Combined | Disruptive | Rare | **0.0043** | 0.1328 | 0.2593 | 1.7571 | 0.0287 | 0.0504 |
| Combined | NS_strict_ | Singleton | 0.1543 | 0.7456 | 0.9860 | 1.1782 | 0.1137 | 0.1339 |
| Combined | NS_strict_ | Rare | 0.2485 | 0.8941 | 0.9990 | 1.0936 | 0.2560 | 0.2800 |
| Combined | NS_broad_ | Singleton | 0.1806 | 0.8043 | 0.9921 | 1.0776 | 0.5132 | 0.5530 |
| Combined | NS_broad_ | Rare | 0.5765 | 0.9954 | 1.0000 | 0.9807 | 1.6119 | 1.5809 |

^a^Schizophrenia (SCZ); bipolar disorder (BD); recurrent major depressive disorder (rMDD); Combined Cases (Combined).

^b^Non-synonymous strictly damaging mutations (NS_strict_); Non-synonymous broadly damaging mutations (NS_broad_).

**Bold**, *P*<0.05; Case/Control Ratio is calculated using Case Rate divided by Control Rate.

**Supplementary Table S11. Gene-Wide Burden Analysis of Rare Functional Variants in DISC1 Regulome for Case-Control Traits**

See: “S11.CaseControlGeneRegulome” sheet in supplementary tables.xlsx

^a^Schizophrenia (SCZ); bipolar disorder (BD); recurrent major depressive disorder (rMDD); Combined Cases (Combined).

^b^Non-synonymous strictly damaging mutations (NS_strict_); Non-synonymous broadly damaging mutations (NS_broad_).

**Bold**, *P*<0.05. Tests are sorted in ascending order of the unadjusted *P*-value. The odds ratio (OR) and standard error (SE).

**Supplementary Table S12. Translin-Associated Factor X Interacting Protein 1 (*TSNAXIP1*) Rare Mutations**

| **Mutation^a^** | **Exon^a^** | **Chr** | **Pos (hg18)** | **Ref base** | **Alt base** | **dbSNP144** | **1000G-Eur Frequency** | **Case MAF** | **Control MAF** | **SCZ^b^** | **rMDD^b^** | **BD^b^** | **Control^b^** |
| --- | --- | --- | --- | --- | --- | --- | --- | --- | --- | --- | --- | --- | --- |
| R46X* | exon2 | 16 | 66405794 | C | T | rs146214814 | 0.0030 | 0.0054 | 0.0041 | 0/6/200 | 0/0/163 | 0/0/189 | 0/7/854 |
| S60Y* | exon3 | 16 | 66412274 | C | A | NA | NA | 0.0009 | 0.0000 | 0/0/200 | 0/1/156 | 0/0/180 | 0/0/843 |
| R75Q | exon3 | 16 | 66412319 | G | A | rs761157513 | NA | 0.0000 | 0.0006 | 0/0/194 | 0/0/157 | 0/0/177 | 0/1/837 |
| R95C | exon4 | 16 | 66412525 | C | T | rs747610440 | NA | 0.0010 | 0.0000 | 0/1/181 | 0/0/148 | 0/0/157 | 0/0/814 |
| D246G | exon7 | 16 | 66416599 | A | G | rs74684664 | 0.0050 | 0.0035 | 0.0052 | 0/1/210 | 0/2/166 | 0/1/191 | 0/9/862 |
| L349P | exon9 | 16 | 66417382 | T | C | rs150340970 | NA | 0.0000 | 0.0006 | 0/0/205 | 0/0/158 | 0/0/179 | 0/1/860 |
| R374W | exon9 | 16 | 66417456 | C | T | rs61999337 | 0.0010 | 0.0009 | 0.0000 | 0/1/198 | 0/0/156 | 0/0/181 | 0/0/846 |
| Q402L* | exon10 | 16 | 66417616 | A | T | rs201147559 | 0.0010 | 0.0010 | 0.0000 | 0/0/178 | 0/1/140 | 0/0/163 | 0/0/812 |
| P432L* | exon11 | 16 | 66417860 | C | T | rs763142409 | NA | 0.0009 | 0.0000 | 0/1/209 | 0/0/164 | 0/0/190 | 0/0/868 |
| R436W* | exon11 | 16 | 66417871 | C | T | rs758788310 | NA | 0.0018 | 0.0000 | 0/2/207 | 0/0/165 | 0/0/189 | 0/0/869 |
| R519Q | exon13 | 16 | 66418368 | G | A | rs140006528 | NA | 0.0036 | 0.0006 | 0/1/205 | 0/1/163 | 0/2/181 | 0/1/867 |
| P605S | exon15 | 16 | 66418898 | C | T | rs146474803 | NA | 0.0000 | 0.0012 | 0/0/210 | 0/0/166 | 0/0/192 | 0/2/865 |
| Splice* | exon16 | 16 | 66419156 | G | C | NA | NA | 0.0009 | 0.0000 | 0/0/210 | 0/1/163 | 0/0/189 | 0/0/868 |
| G645D | exon16 | 16 | 66419192 | G | A | rs190858166 | NA | 0.0009 | 0.0000 | 0/0/211 | 0/1/164 | 0/0/192 | 0/0/870 |
| S671L | exon16 | 16 | 66419270 | C | T | rs763671210 | NA | 0.0009 | 0.0000 | 0/1/209 | 0/0/166 | 0/0/189 | 0/0/871 |
| R694W | exon16 | 16 | 66419338 | C | T | rs140308084 | NA | 0.0000 | 0.0006 | 0/0/210 | 0/0/166 | 0/0/189 | 0/1/869 |
| R702C* | exon16 | 16 | 66419362 | C | T | rs150288850 | NA | 0.0009 | 0.0000 | 0/0/211 | 0/1/166 | 0/0/190 | 0/0/871 |

*Non-synonymous strict damaging mutations (NS_strict_): disruptive plus missense variants predicted as damaging by all five algorithms (PolyPhen2 HumDiv and HumVar, SIFT, LRT and MutationTaster)

^a^Variant annotation is based on NM_001288990.

^b^Genotype counts are shown for Schizophrenia (SCZ), bipolar disorder (BD), recurrent major depressive disorder (rMDD) cases and the Lothian Birth Cohort of 1936 controls (CTL).

**Supplementary Table S13. Gene Set Burden Analysis of Rare Functional Variants in the DISC1 Interactome for Quantitative Traits**

| **Trait^a^** | **Mutation Class^b^** | **MAFbin** | **N.SNP.Test** | **Unadjusted *P*** | **FWER_within_ *P*** | **FWER_cross_ *P*** | **Beta** | **SE** |
| --- | --- | --- | --- | --- | --- | --- | --- | --- |
| ***Cognitive Measures*** | |  |  |  |  |  |  |  |
| MHT11 | Disruptive | Singleton | 10 | **9.35E-05** | **0.0005** | **0.0043** | -7.1141 | 3.6863 |
| MHT11 | Disruptive | Rare | 18 | 0.0541 | 0.2367 | 0.8785 | -2.9891 | 2.2262 |
| MHT11 | NS_strict_ | Singleton | 76 | **0.0003** | **0.0017** | **0.0122** | -2.7865 | 1.2877 |
| MHT11 | NS_strict_ | Rare | 126 | **0.0326** | 0.1564 | 0.7266 | -0.9314 | 0.7755 |
| MHT11 | NS_broad_ | Singleton | 344 | **0.0447** | 0.2043 | 0.8296 | -0.8037 | 0.5970 |
| MHT11 | NS_broad_ | Rare | 595 | 0.5013 | 0.9187 | 1.0000 | 0.0223 | 0.3087 |
| MHT70 | Disruptive | Singleton | 9 | **0.0056** | **0.0294** | 0.2209 | -6.6785 | 2.7886 |
| MHT70 | Disruptive | Rare | 17 | 0.3384 | 0.7995 | 0.9999 | -1.5651 | 1.6838 |
| MHT70 | NS_strict_ | Singleton | 77 | **0.0287** | 0.1354 | 0.6835 | -2.3177 | 0.9725 |
| MHT70 | NS_strict_ | Rare | 128 | 0.0994 | 0.3882 | 0.9728 | -0.9408 | 0.5864 |
| MHT70 | NS_broad_ | Singleton | 354 | 0.3898 | 0.8460 | 0.9999 | -0.4608 | 0.4516 |
| MHT70 | NS_broad_ | Rare | 610 | 0.6196 | 0.9665 | 1.0000 | 0.2453 | 0.2334 |
| MHT70-11 | Disruptive | Singleton | 9 | 0.5837 | 0.9563 | 1.0000 | -1.7825 | 1.8772 |
| MHT70-11 | Disruptive | Rare | 17 | 0.9126 | 0.9997 | 1.0000 | 0.1731 | 1.1339 |
| MHT70-11 | NS_strict_ | Singleton | 75 | 0.4291 | 0.8770 | 1.0000 | -0.7946 | 0.6551 |
| MHT70-11 | NS_strict_ | Rare | 125 | 0.9219 | 0.9997 | 1.0000 | -0.1955 | 0.3950 |
| MHT70-11 | NS_broad_ | Singleton | 340 | 0.3724 | 0.8309 | 1.0000 | 0.0603 | 0.3041 |
| MHT70-11 | NS_broad_ | Rare | 591 | 0.9444 | 0.9998 | 1.0000 | 0.3243 | 0.1573 |
| NART | Disruptive | Singleton | 10 | **0.0051** | **0.0289** | 0.2012 | -6.9970 | 2.6307 |
| NART | Disruptive | Rare | 18 | 0.2523 | 0.6872 | 0.9998 | -2.3257 | 1.5884 |
| NART | NS_strict_ | Singleton | 78 | 0.0874 | 0.3417 | 0.9606 | -2.2643 | 0.9175 |
| NART | NS_strict_ | Rare | 129 | 0.4730 | 0.9036 | 1.0000 | -0.9683 | 0.5532 |
| NART | NS_broad_ | Singleton | 356 | 0.5297 | 0.9318 | 1.0000 | -0.1771 | 0.4260 |
| NART | NS_broad_ | Rare | 612 | 0.5258 | 0.9291 | 1.0000 | -0.0910 | 0.2202 |
| Gf | Disruptive | Singleton | 10 | **0.0293** | 0.1378 | 0.6911 | -0.5152 | 0.3097 |
| Gf | Disruptive | Rare | 18 | 0.3673 | 0.8262 | 0.9999 | -0.1573 | 0.1870 |
| Gf | NS_strict_ | Singleton | 75 | 0.1554 | 0.5186 | 0.9966 | -0.1703 | 0.1080 |
| Gf | NS_strict_ | Rare | 126 | 0.1551 | 0.5184 | 0.9965 | -0.1002 | 0.0651 |
| Gf | NS_broad_ | Singleton | 350 | 0.4616 | 0.8995 | 1.0000 | -0.0449 | 0.0502 |
| Gf | NS_broad_ | Rare | 605 | 0.1938 | 0.5891 | 0.9991 | -0.0046 | 0.0259 |
| ***Psychiatric Symptoms*** | |  |  |  |  |  |  |  |
| Neuroticism | Disruptive | Singleton | 8 | **0.0154** | 0.0754 | 0.4765 | 6.5671 | 2.2564 |
| Neuroticism | Disruptive | Rare | 15 | 0.1581 | 0.5215 | 0.9969 | 1.7280 | 1.3624 |
| Neuroticism | NS_strict_ | Singleton | 68 | 0.1688 | 0.5429 | 0.9978 | 0.2127 | 0.7869 |
| Neuroticism | NS_strict_ | Rare | 117 | 0.2502 | 0.6863 | 0.9998 | -0.2497 | 0.4745 |
| Neuroticism | NS_broad_ | Singleton | 320 | 0.3592 | 0.8204 | 0.9999 | 0.2554 | 0.3654 |
| Neuroticism | NS_broad_ | Rare | 569 | 0.4616 | 0.8978 | 1.0000 | -0.3550 | 0.1889 |
| Anxiety | Disruptive | Singleton | 10 | 0.5153 | 0.9235 | 1.0000 | 2.0461 | 0.9927 |
| Anxiety | Disruptive | Rare | 18 | 0.6085 | 0.9614 | 1.0000 | 0.3739 | 0.5994 |
| Anxiety | NS_strict_ | Singleton | 78 | 0.3098 | 0.7584 | 0.9999 | 0.8798 | 0.3462 |
| Anxiety | NS_strict_ | Rare | 129 | **0.0349** | 0.1561 | 0.7500 | 0.1394 | 0.2087 |
| Anxiety | NS_broad_ | Singleton | 356 | 0.6432 | 0.9713 | 1.0000 | 0.2508 | 0.1607 |
| Anxiety | NS_broad_ | Rare | 612 | 0.2737 | 0.7060 | 0.9999 | -0.0917 | 0.0831 |
| Depression | Disruptive | Singleton | 10 | 0.4476 | 0.8900 | 0.9999 | 0.4975 | 0.7151 |
| Depression | Disruptive | Rare | 18 | 0.1088 | 0.4023 | 0.9800 | 0.1265 | 0.4318 |
| Depression | NS_strict_ | Singleton | 77 | 0.2971 | 0.7513 | 0.9999 | 0.2985 | 0.2494 |
| Depression | NS_strict_ | Rare | 128 | 0.7048 | 0.9842 | 1.0000 | 0.1120 | 0.1504 |
| Depression | NS_broad_ | Singleton | 355 | **0.0431** | 0.1911 | 0.8167 | 0.2587 | 0.1158 |
| Depression | NS_broad_ | Rare | 611 | 0.7032 | 0.9840 | 1.0000 | 0.0492 | 0.0599 |

^a^Moray House Test at age 11 (MHT11); Moray House Test at age 70 (MHT70); Moray House Test at age 70 adjusted for the Moray House Test score at age 11 (MHT70-11); National Adult Reading Test (NART); General Fluid Intelligence (Gf); Hospital Anxiety Depression Scales - Depression (Depression); Hospital Anxiety Depression Scales - Anxiety (Anxiety); NEO Five-Factor Inventory Neuroticism (Neuroticism).

^b^Non-synonymous strictly damaging mutations (NS_strict_); Non-synonymous broadly damaging mutations (NS_broad_).

**Bold**, *P*<0.05. The effect size (Beta) and standard error (SE).

**Supplementary Table S14. Gene-Wide Burden Analysis of Rare Functional Variants in the DISC1 Interactome for Quantitative Traits**

See: “S14.QuantitativeGeneInteractome” sheet in supplementary tables.xlsx

^a^Moray House Test at age 11 (MHT11); Moray House Test at age 70 (MHT70); Moray House Test at age 70 adjusted for the Moray House Test score at age 11 (MHT70-11); National Adult Reading Test (NART); General Fluid Intelligence (Gf); Hospital Anxiety Depression Scales - Depression (Depression); Hospital Anxiety Depression Scales - Anxiety (Anxiety); NEO Five-Factor Inventory Neuroticism (Neuroticism).

^b^Non-synonymous strictly damaging mutations (NS_strict_); Non-synonymous broadly damaging mutations (NS_broad_).

**Bold**, *P*<0.05. Tests are sorted in ascending order of the unadjusted *P*-value. The effect size (Beta) and standard error (SE).

**Supplementary Table S15. Gene Set Burden Analysis of Rare Functional Variants in the DISC1 Regulome for Quantitative Traits**

| **Trait^a^** | **Mutation Class^b^** | **MAFbin** | **N.SNP.Test** | **Unadjusted *P*** | **FWER_within_ *P*** | **FWER_cross_ *P*** | **Beta** | **SE** |
| --- | --- | --- | --- | --- | --- | --- | --- | --- |
| ***Cognitive Measures*** | |  |  |  |  |  |  |  |
| MHT11 | Disruptive | Singleton | 8 | 0.9765 | 1.0000 | 1.0000 | 3.6344 | 4.1177 |
| MHT11 | Disruptive | Rare | 14 | 0.9593 | 0.9999 | 1.0000 | 0.4846 | 2.3509 |
| MHT11 | NS_strict_ | Singleton | 93 | 0.5367 | 0.9357 | 1.0000 | -0.6650 | 1.1353 |
| MHT11 | NS_strict_ | Rare | 141 | 0.4442 | 0.8861 | 1.0000 | -0.8979 | 0.7527 |
| MHT11 | NS_broad_ | Singleton | 428 | 0.4343 | 0.8794 | 1.0000 | -0.6673 | 0.5190 |
| MHT11 | NS_broad_ | Rare | 719 | 0.3258 | 0.7787 | 1.0000 | -0.5767 | 0.2916 |
| MHT70 | Disruptive | Singleton | 8 | 0.8481 | 0.9979 | 1.0000 | 5.3886 | 3.1132 |
| MHT70 | Disruptive | Rare | 14 | 0.7998 | 0.9950 | 1.0000 | 2.4608 | 1.7762 |
| MHT70 | NS_strict_ | Singleton | 99 | **0.0014** | **0.0079** | 0.0609 | -1.7895 | 0.8596 |
| MHT70 | NS_strict_ | Rare | 146 | **0.0142** | 0.0688 | 0.4392 | -1.1343 | 0.5704 |
| MHT70 | NS_broad_ | Singleton | 444 | **0.0093** | **0.0451** | 0.3160 | -0.7885 | 0.3927 |
| MHT70 | NS_broad_ | Rare | 736 | 0.1417 | 0.4727 | 0.9942 | -0.4136 | 0.2205 |
| MHT70-11 | Disruptive | Singleton | 8 | 0.8646 | 0.9977 | 1.0000 | 3.2670 | 2.0973 |
| MHT70-11 | Disruptive | Rare | 14 | 0.2644 | 0.6969 | 0.9999 | 2.2696 | 1.1966 |
| MHT70-11 | NS_strict_ | Singleton | 93 | **0.0131** | 0.0657 | 0.4143 | -1.4338 | 0.5777 |
| MHT70-11 | NS_strict_ | Rare | 140 | 0.3453 | 0.7940 | 1.0000 | -0.5199 | 0.3834 |
| MHT70-11 | NS_broad_ | Singleton | 425 | **0.0014** | **0.0075** | 0.0617 | -0.3175 | 0.2644 |
| MHT70-11 | NS_broad_ | Rare | 715 | **0.0280** | 0.1294 | 0.6687 | -0.0010 | 0.1485 |
| NART | Disruptive | Singleton | 8 | 0.6397 | 0.9706 | 1.0000 | 3.6591 | 2.9369 |
| NART | Disruptive | Rare | 14 | 0.9836 | 1.0000 | 1.0000 | 1.1720 | 1.6756 |
| NART | NS_strict_ | Singleton | 99 | 0.2747 | 0.7099 | 1.0000 | -0.6962 | 0.8109 |
| NART | NS_strict_ | Rare | 147 | **0.0457** | 0.1978 | 0.8297 | -0.9724 | 0.5381 |
| NART | NS_broad_ | Singleton | 447 | 0.1256 | 0.4341 | 0.9892 | -0.5452 | 0.3704 |
| NART | NS_broad_ | Rare | 740 | 0.3995 | 0.8455 | 1.0000 | -0.4607 | 0.2080 |
| Gf | Disruptive | Singleton | 8 | 0.4882 | 0.9138 | 1.0000 | 0.2448 | 0.3458 |
| Gf | Disruptive | Rare | 14 | 0.4246 | 0.8753 | 1.0000 | 0.0172 | 0.1973 |
| Gf | NS_strict_ | Singleton | 95 | 0.3744 | 0.8327 | 1.0000 | -0.0947 | 0.0955 |
| Gf | NS_strict_ | Rare | 143 | 0.2384 | 0.6650 | 0.9999 | -0.0957 | 0.0634 |
| Gf | NS_broad_ | Singleton | 440 | 0.1647 | 0.5327 | 0.9976 | -0.0385 | 0.0436 |
| Gf | NS_broad_ | Rare | 732 | 0.3885 | 0.8469 | 1.0000 | -0.0119 | 0.0245 |
| ***Psychiatric Symptoms*** | |  |  |  |  |  |  |  |
| Neuroticism | Disruptive | Singleton | 7 | 0.2730 | 0.7135 | 1.0000 | -2.6295 | 2.5190 |
| Neuroticism | Disruptive | Rare | 13 | 0.3603 | 0.8151 | 1.0000 | -0.2165 | 1.4372 |
| Neuroticism | NS_strict_ | Singleton | 91 | 0.8691 | 0.9993 | 1.0000 | 0.3354 | 0.6955 |
| Neuroticism | NS_strict_ | Rare | 136 | 0.8089 | 0.9952 | 1.0000 | 0.1261 | 0.4616 |
| Neuroticism | NS_broad_ | Singleton | 400 | 0.8478 | 0.9982 | 1.0000 | -0.4638 | 0.3177 |
| Neuroticism | NS_broad_ | Rare | 682 | 0.8925 | 0.9997 | 1.0000 | -0.2167 | 0.1784 |
| Anxiety | Disruptive | Singleton | 8 | 0.4673 | 0.9006 | 1.0000 | -2.2912 | 1.1082 |
| Anxiety | Disruptive | Rare | 14 | 0.7084 | 0.9844 | 1.0000 | -0.4895 | 0.6323 |
| Anxiety | NS_strict_ | Singleton | 99 | 0.6367 | 0.9711 | 1.0000 | 0.3305 | 0.3060 |
| Anxiety | NS_strict_ | Rare | 147 | 0.9529 | 1.0000 | 1.0000 | 0.1551 | 0.2031 |
| Anxiety | NS_broad_ | Singleton | 447 | 0.5648 | 0.9486 | 1.0000 | 0.0332 | 0.1398 |
| Anxiety | NS_broad_ | Rare | 740 | 0.6768 | 0.9809 | 1.0000 | -0.0890 | 0.0785 |
| Depression | Disruptive | Singleton | 8 | 0.9594 | 1.0000 | 1.0000 | -0.3317 | 0.7984 |
| Depression | Disruptive | Rare | 14 | 0.9879 | 1.0000 | 1.0000 | -0.1067 | 0.4555 |
| Depression | NS_strict_ | Singleton | 98 | 0.6834 | 0.9812 | 1.0000 | 0.3950 | 0.2204 |
| Depression | NS_strict_ | Rare | 146 | 0.6177 | 0.9663 | 1.0000 | 0.2661 | 0.1463 |
| Depression | NS_broad_ | Singleton | 446 | 0.9579 | 0.9999 | 1.0000 | -0.0391 | 0.1007 |
| Depression | NS_broad_ | Rare | 738 | 0.9988 | 1.0000 | 1.0000 | -0.0760 | 0.0565 |

^a^Moray House Test at age 11 (MHT11); Moray House Test at age 70 (MHT70); Moray House Test at age 70 adjusted for the Moray House Test score at age 11 (MHT70-11); National Adult Reading Test (NART); General Fluid Intelligence (Gf); Hospital Anxiety Depression Scales - Depression (Depression); Hospital Anxiety Depression Scales - Anxiety (Anxiety); NEO Five-Factor Inventory Neuroticism (Neuroticism).

^b^Non-synonymous strictly damaging mutations (NS_strict_); Non-synonymous broadly damaging mutations (NS_broad_).

**Bold**, *P*<0.05. Tests are sorted in ascending order of the unadjusted *P*-value. The effect size (Beta) and standard error (SE).

**Supplementary Table S16. Gene-Wide Burden Analysis of Rare Functional Variants in the DISC1 Regulome for Quantitative Traits**

See: “S16.QuantitativeGeneRegulome” sheet in supplementary tables.xlsx

^a^Moray House Test at age 11 (MHT11); Moray House Test at age 70 (MHT70); Moray House Test at age 70 adjusted for the Moray House Test score at age 11 (MHT70-11); National Adult Reading Test (NART); General Fluid Intelligence (Gf); Hospital Anxiety Depression Scales - Depression (Depression); Hospital Anxiety Depression Scales - Anxiety (Anxiety); NEO Five-Factor Inventory Neuroticism (Neuroticism).

^b^Non-synonymous strictly damaging mutations (NS_strict_); Non-synonymous broadly damaging mutations (NS_broad_).

**Bold**, *P*<0.05. Tests are sorted in ascending order of the unadjusted *P*-value. The effect size (Beta) and standard error (SE).

**Supplementary Table S17. Gene Ontology Enrichment Analyses of the DISC1 Interactome**

See: “S17.GeneOntologyInteractome” sheet in supplementary tables.xlsx

**Supplementary Table S18. Gene Ontology Enrichment Analyses of the DISC1 Regulome**

See: “S18.GeneOntologyRegulome” sheet in supplementary tables.xlsx

**Supplementary Figures**

**Supplementary Figure S1: Distribution of Coverage in Samples**


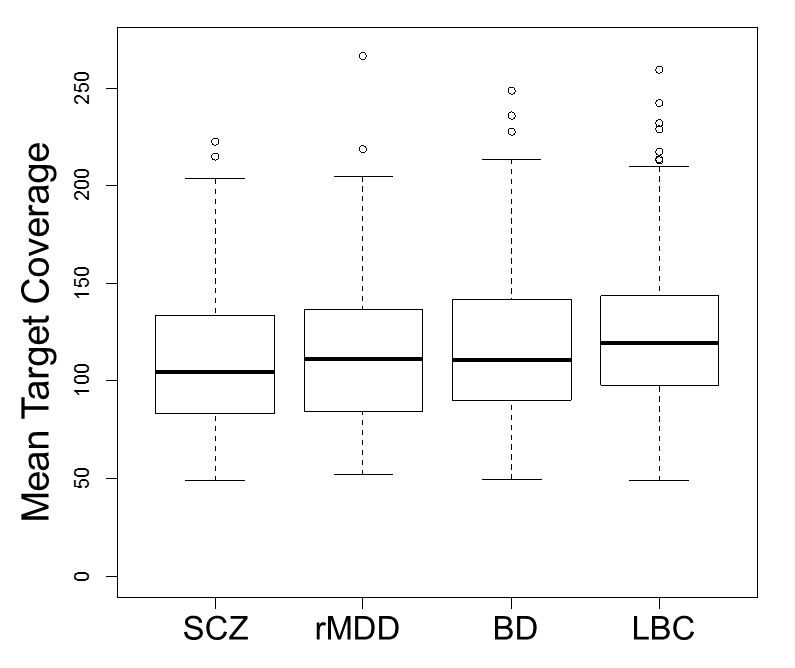


The distribution of the mean target coverage for samples with 80% of the targets at ≥20x depth in schizophrenia (SCZ), recurrent major depressive disorder (rMDD), bipolar disorder (BD) and the Lothian Birth Cohort of 1936 (LBC1936). No evidence of sequencing bias was observed between cases (SCZ, BD and rMDD) and controls (LBC1936).

**Supplementary Figure S2: A Flowchart of Quality Control**


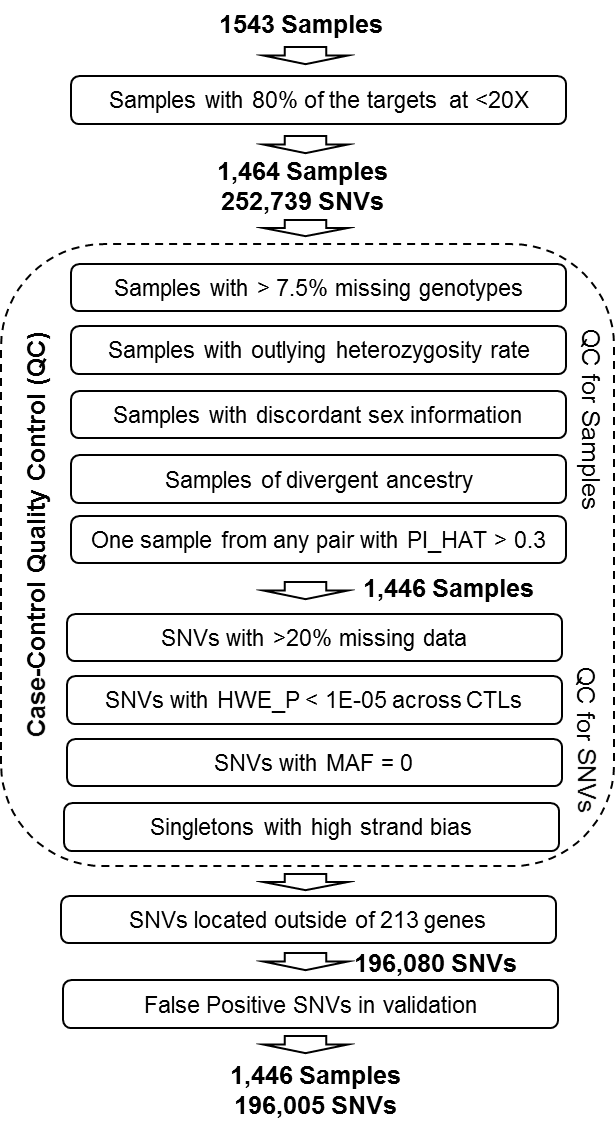


Flowchart depicting the filtering strategy involved in removal of outlying samples and single nucleotide variants (SNVs) that introduce bias (rounded rectangles).

**Supplementary Figure S3: Multidimensional Scaling Plots**


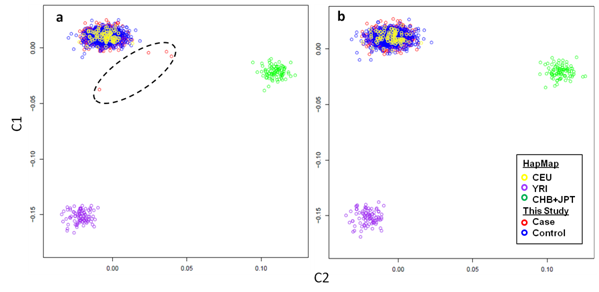


Multidimensional scaling (MDS) plots for the samples in the (a) raw dataset and (b) final dataset. The four samples (within dashed circle) which clustered away from the HapMap European population (CEU, yellow) were removed from further analysis.

**Supplementary Figure S4: Missing Variant Rate versus Heterozygosity Rate across all Samples**


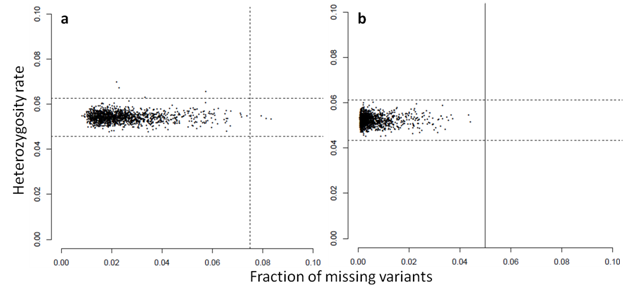


Missing variant rate versus heterozygosity rate across all samples in the (a) raw dataset and b) final dataset. The samples with a missing variant rate>0.75 (vertical dashed line) and/or a heterozygosity rate ± 4 standard deviation from the mean (horizontal dashed lines) were excluded from further analysis. These samples usually have low DNA quality or concentration.^28^

**Supplementary Figure S5: Missing Sample Rate versus Hardy-Weinberg equilibrium *P*-values across all SNVs**


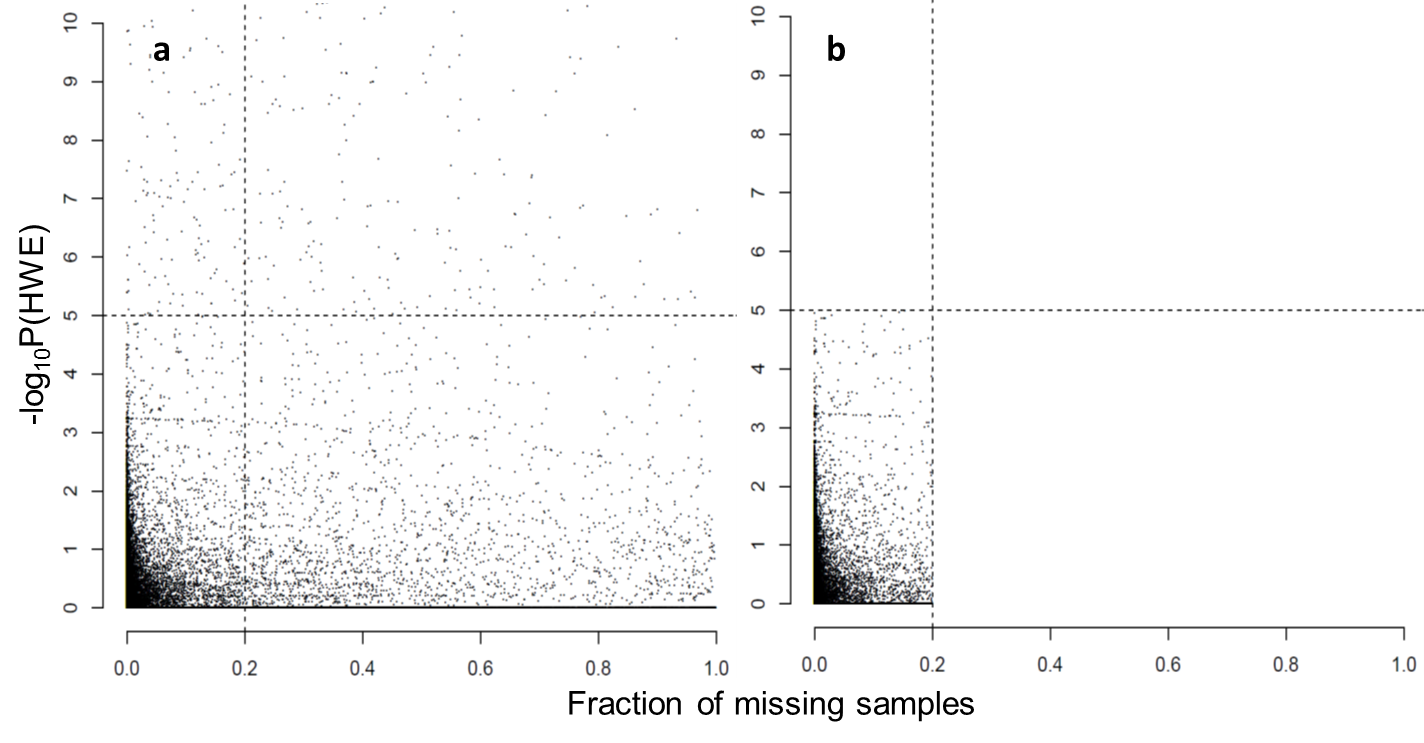


Missing sample rate versus Hardy-Weinberg equilibrium (HWE) *P*-values across all SNVs in the (a) raw dataset and (b) final dataset. The SNVs with a missing sample rate>0.2 (vertical dashed line) and/or Hardy-Weinberg equilibrium *P*-values<0.00001 (horizontal dashed lines) in controls were excluded from further analysis. These variants represent false positives and genotyping errors.^28^

**Supplementary Figure S6: Minor Allele Frequencies in the 1000 Genomes Project and LBC1936 controls**


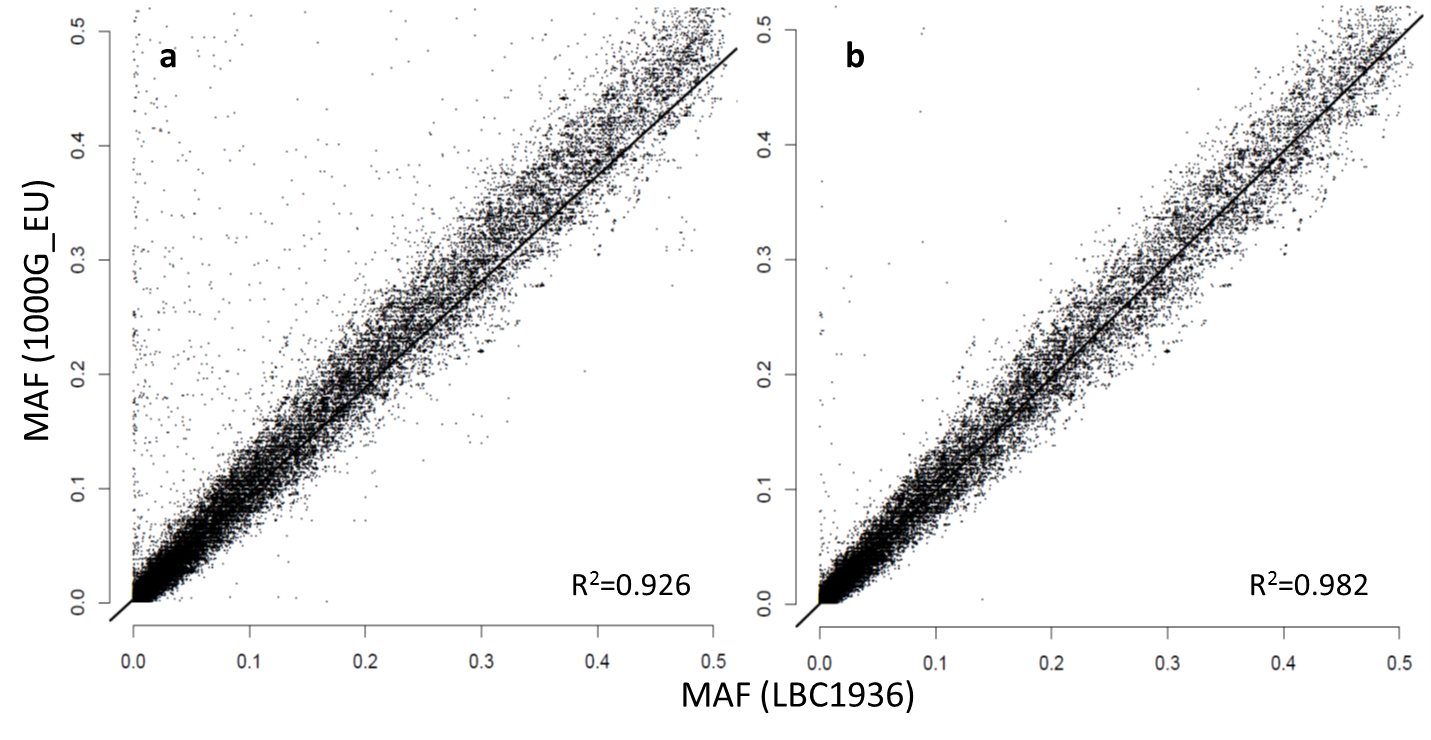


Minor allele frequencies (MAFs) in the 1000 Genomes Project (2015 Aug annotations) and LBC1936 controls in the (a) raw dataset and (b) final dataset. The variant MAFs identified in the European subset of the 1000 Genomes Project (1000G_EU) versus healthy controls (LBC1936) data set are more correlated after applying quality control filters (R^2^=0.982).

**Supplementary Figure S7: Quantile-Quantile Plots of Gene-wide Burden Analysis of Rare Damaging Mutations for Case-Control Traits**

**Supplementary Figure S8: Quantile-Quantile Plots of Gene-wide Burden Analysis of Rare Damaging Mutations for Quantitative Traits**

**Supplementary Figure S9: Gene Ontology Enrichment Analyses for Biological Process**

See: Supplementary Figure S9 in Supplementary Figures S9-11.pptx

**Supplementary Figure S10: Gene Ontology Enrichment Analyses for Molecular Function**

See: Supplementary Figure S10 in Supplementary Figures S9-11.pptx

**Supplementary Figure S11: Gene Ontology Enrichment Analyses for Cellular Component**

See: Supplementary Figure S11 in Supplementary Figures S9-11.pptx
